# Murine leukemia virus p12 tethers the capsid-containing pre-integration complex to chromatin by binding directly to host nucleosomes in mitosis

**DOI:** 10.1371/journal.ppat.1007117

**Published:** 2018-06-15

**Authors:** Madushi Wanaguru, David J. Barry, Donald J. Benton, Nicola J. O’Reilly, Kate N. Bishop

**Affiliations:** 1 Retroviral Replication Laboratory, The Francis Crick Institute, London, United Kingdom; 2 Advanced Light Microscopy Facility, The Francis Crick Institute, London, United Kingdom; 3 Structural Biology of Disease Processes Laboratory, The Francis Crick Institute, London, United Kingdom; 4 Peptide Chemistry, The Francis Crick Institute, London, United Kingdom; Johns Hopkins, UNITED STATES

## Abstract

The murine leukaemia virus (MLV) Gag cleavage product, p12, is essential for both early and late steps in viral replication. The N-terminal domain of p12 binds directly to capsid (CA) and stabilises the mature viral core, whereas defects in the C-terminal domain (CTD) of p12 can be rescued by addition of heterologous chromatin binding sequences (CBSs). We and others hypothesised that p12 tethers the pre-integration complex (PIC) to host chromatin ready for integration. Using confocal microscopy, we have observed for the first time that CA localises to mitotic chromatin in infected cells in a p12-dependent manner. GST-tagged p12 alone, however, did not localise to chromatin and mass-spectrometry analysis of its interactions identified only proteins known to bind the p12 region of Gag. Surprisingly, the ability to interact with chromatin was conferred by a single amino acid change, M63I, in the p12 CTD. Interestingly, GST-p12_M63I showed increased phosphorylation in mitosis relative to interphase, which correlated with an increased interaction with mitotic chromatin. Mass-spectrometry analysis of GST-p12_M63I revealed nucleosomal histones as primary interactants. Direct binding of MLV p12_M63I peptides to histones was confirmed by biolayer-interferometry (BLI) assays using highly-avid recombinant poly-nucleosomal arrays. Excitingly, using this method, we also observed binding between MLV p12_WT and nucleosomes. Nucleosome binding was additionally detected with p12 orthologs from feline and gibbon ape leukemia viruses using both pull-down and BLI assays, indicating that this a common feature of gammaretroviral p12 proteins. Importantly, p12 peptides were able to block the binding of the prototypic foamy virus CBS to nucleosomes and *vice versa*, implying that their docking sites overlap and suggesting a conserved mode of chromatin tethering for different retroviral genera. We propose that p12 is acting in a similar capacity to CPSF6 in HIV-1 infection by facilitating initial chromatin targeting of CA-containing PICs prior to integration.

## Author summary

In addition to matrix, capsid and nucleocapsid, the Gag polyproteins of many retroviruses also encode additional cleavage products, such as the p12 protein of murine leukemia virus. p12 is essential for both early and late replication events, but its function during early infection remains poorly characterised. Recent work has shown that the N-terminal domain of p12 binds to and stabilises the capsid shell that surrounds the viral core. Defects in the C-terminal domain of p12 prevented the viral pre-integration complex (PIC) from localising to chromatin during infection, and this could be rescued by the addition of a heterologous chromatin binding domain. In this study, we show that p12 is able to tether viral PICs containing capsid to mitotic chromatin by directly binding both hexameric capsid and nucleosomal histone proteins. Additionally, the affinity of p12 for chromatin may be enhanced by its binding to capsid and by its phosphorylation in mitosis. Excitingly, the mechanism of nucleosome binding appears to be conserved between gammaretroviruses and spumaretroviruses. Furthermore, influencing global nuclear targeting of the PIC by linking capsid to chromatin is reminiscent of the proposed role of the capsid-binding host protein, CPSF6, in HIV-1 infection.

## Introduction

**Fig 1:**
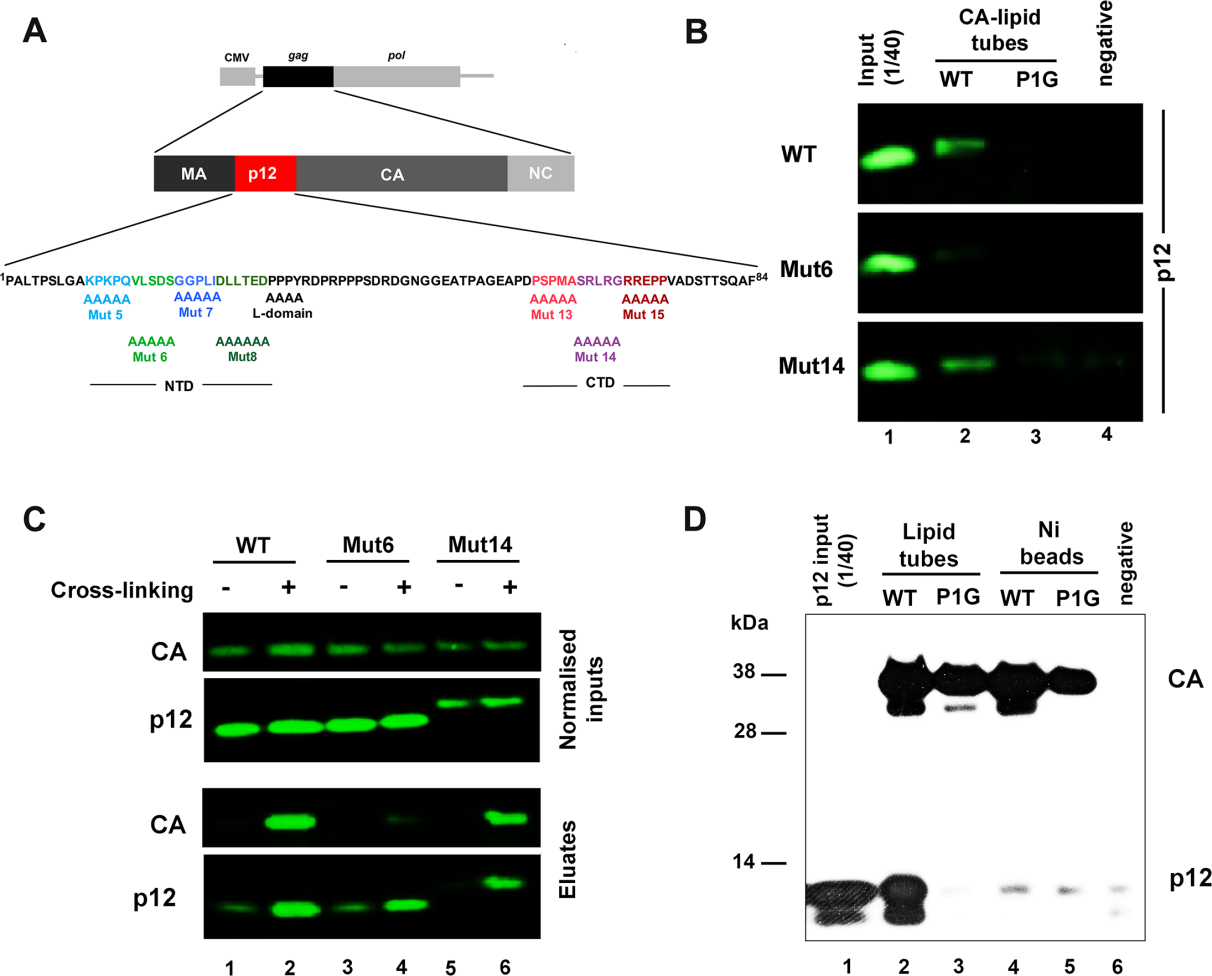
The N-terminal domain of MLV p12 directly binds hexameric CA arrays. (A) Schematic representation of the MLV Gag-Pol expression plasmid used in this study, showing the Mo-MLV p12 sequence with alanine substitution mutants below. (B) Immunoblot showing binding of p12 to N-MLV CA. His-tagged WT (lane 2) or P1G mutant (lane 3) CA proteins were immobilised on lipid nanotubes comprising the Ni^2+-^chelating lipid, DGS-NTA, prior to incubation with purified p12_WT, p12_mut6 (NTD mutant) or p12_mut14 (CTD mutant). No lipid tubes or CA were included in the negative control reactions (lane 4). CA nanotubes were pelleted, and any bound p12 was revealed by SDS-PAGE and immunoblotting with a rabbit anti-p12 antibody. 1/40^th^ of the purified p12 protein input was loaded in lane 1. (C) Co-immunoprecipitation (Co-IP) of CA with p12 from viral lysates. Mo-MLV virus-like particles (VLPs) carrying myc-tagged p12_WT (lanes 1 and 2), p12_mut6 (lanes 3 and 4) or p12_mut14 (lanes 5 and 6) were either untreated (lanes 1, 3 and 5) or fixed in 1% formaldehyde (lanes 2, 4 and 6) prior to lysis. Myc-p12 was immunoprecipitated from normalised viral lysates using an anti-myc antibody. 1/50^th^ of input lysates and 1/5^th^ of Co-IP elutions were analysed by immunoblotting with anti-CA and rabbit anti-p12 antibodies. (D) Immunoblot showing binding of p12 specifically to CA hexamers. His-tagged WT (lanes 2 and 4) or P1G mutant (lanes 3 and 5) CA proteins were immobilised as hexamers on lipid nanotubes (lanes 2 and 3), or randomly on Ni-NTA beads (lanes 4 and 5). No CA, beads or tubes were included in the negative control (lane 6). After incubation with p12, the CA assemblies were pelleted and analysed by SDS-PAGE and immunoblotting for CA (anti-His) and p12 (mouse anti-p12). 1/40^th^ of the purified p12 protein input was loaded in lane 1.

The retroviral Gag polyprotein plays essential roles at multiple stages of the viral life cycle. In late infection, Gag mediates the assembly and release of progeny viral particles. It is then proteolyically cleaved during viral maturation into a number of individual proteins. In addition to the three main structural proteins, matrix (MA), capsid (CA) and nucleocapsid (NC), many retroviruses also produce additional Gag cleavage products such as the p12 protein of MLV and the p6 protein of HIV-1 [1]. The proline-rich late (L)-domains which allow Gag to recruit the cellular ESCRT machinery required for efficient virus budding are frequently found in these additional Gag cleavage products (Fig 1A) [2–5]. However, it is likely that these proteins also have other functions, for example, MLV p12 is known to be essential for early replication events.

Initially, a subset of alanine-scanning substitution mutations in Moloney (Mo)-MLV p12 were shown to inhibit replication of MLV, predominantly at a stage post reverse transcription [6]. These mutations clustered into two regions on p12, one located towards the 5’ and the other towards the 3’ end of the gene. These two groups of mutants were later shown to be phenotypically distinct from each other and two functional domains within the protein were defined, both of which were conserved among p12 orthologues of other gammaretroviruses [7]. Furthermore, infections with mixed particle containing both wild type (WT) and mutant viruses revealed that the two domains act sequentially in the virus life cycle, with the N-terminal function required before that of the C-terminus [7].

Functional cooperativity between p12 and CA was first demonstrated in a study of chimeric MLV/ spleen necrosis virus (SNV) particles, with the chimeras being infectious only when p12 (p18 in the case of SNV) was accompanied by CA from the same virus [8]. Recent work from our laboratory has shown that the N-terminal domain (NTD) of p12 (aa 10–30 of Mo-MLV p12) binds to and stabilises the CA lattice [9]. Alterations in p12 do not affect Gag processing, but viral particles carrying mutations in the NTD of p12 (mutants 5–8 in Fig 1A) display core abnormalities and reduced binding to CA-targeting host restriction factors [7, 9]. The precise mechanism by which p12 influences global core stability has not yet been established.

In recent years, a potential role for the p12 C-terminal domain (CTD) (aa 60–74 of Mo-MLV p12) in tethering the pre-integration complex (PIC) to mitotic chromatin has emerged. Mo-MLV p12 has been observed to co-localise with viral DNA in infected cells using fluorescence microscopy [10]. Viral complexes containing p12 initially localise in the cytoplasm, then traffic towards the nucleus and accumulate on mitotic chromosomes [10, 11]. Live cell imaging of GFP-p12 carrying viral particles has further revealed that the association with chromatin is a transient event, with p12 being released upon chromatin decondensation [11]. Importantly, mutant viral particles carrying alterations in the p12 CTD (mutants 13–15 in Fig 1A) are deficient in chromatin docking and replication, but are partially rescued in both tethering and infectivity by the insertion of heterologous chromatin binding modules into p12 [7, 11, 12].

Tethering to chromatin in mitosis is a nuclear retention mechanism shared between different types of viruses. DNA viruses such as gamma-herpesviruses and papillomaviruses use this strategy to maintain their episomal genomes in the host nuclei during cell division [13]. On the other hand, retroviruses must interact with chromatin in order to integrate their genomes as an essential part of their replication. Some retroviruses, including the gammaretrovirus Mo-MLV and the spumavirus prototypic foamy virus (PFV), can only gain access to chromatin during mitosis when the nuclear envelope breaks down, and may therefore initially tether to mitotic chromatin in order to reside in the nucleus once the nuclear membrane has reformed. Viral proteins that mediate chromatin tethering interact with diverse chromatin components. The EBNA-1 protein of the Epstein-Barr virus (EBV) binds both host DNA and EBP2, a chromatin-associated protein, whereas the LANA protein of Kaposi’s sarcoma herpes virus (KSHV) and the chromatin binding sequence (CBS) of PFV Gag both bind directly to nucleosomal histones H2A-H2B [14–18]. It is not known whether p12 directly binds chromatin or if it interacts with another chromatin binding factor. Notably, fluorescence microscopy revealed that recombinant Mo-MLV p12 did not localise to mitotic chromatin in mammalian cells [19]. One possibility is that another viral protein is required for chromatin tethering. For example, binding to CA could alter the conformation of p12 and influence its affinity for chromatin, or the viral integrase (IN) protein could contribute to chromatin tethering of the PIC via its interactions with the bromodomain and extraterminal domain (BET) family of proteins [20–22]. Alternatively, the inability of recombinant Mo-MLV p12 to bind chromatin could be due to the absence of an essential post-translational modification. Previous studies have identified p12 as the main phosphorylated protein in Mo-MLV with the majority of phosphorylation occurring on serine-61 within the CTD [19, 23–25]. In contrast with viral p12, recombinant Mo-MLV p12 was found to be non-phosphorylated in mammalian cells [19]. However, the importance of p12 phosphorylation for viral replication is unclear. Substitution of S61 with both phosphoablative alanine and ‘phosphomimetic’ aspartic acid causes severe defects in Mo-MLV infectivity [19, 25]. Furthermore, viral revertants that arise from live passaging of a p12 mutant, SS(61,65)AA, show near WT levels of infectivity without recovery of phosphorylation [25]. Instead, revertants that maintain the original SS(61,65)AA mutations carry additional changes such as an isoleucine substitution at M63 (M63I) or R/K substitutions at various residues [25]. In general, these compensatory mutations localise either within the p12 CTD region or adjacent to it, suggesting a possible involvement in chromatin binding. In fact, the M63I substitution has been observed to rescue mitotic chromatin tethering of SS(61,65)AA in infected cells [11]. Interestingly, M63I can also rescue chromatin binding of recombinant Mo-MLV in mammalian cells, suggesting that it could be compensating for an absence of phosphorylation in this context [19].

In this study, we characterised the interactions of p12 with both viral and host proteins, using a combination of virological, biochemical and imaging assays. We observed a p12 CTD-dependent association of CA with mitotic chromatin in MLV-infected cells, suggesting that CA is still present when p12 tethers the PIC to chromatin. We also showed that although recombinant GST-tagged WT Mo-MLV p12 shows little detectable chromatin association in a range of cell-based assays, other gammaretroviral p12 proteins do show chromatin interactions, and that subtle mutations can increase the association of Mo-MLV p12 with chromatin. Furthermore, we established that recombinant p12 has a higher affinity for chromatin in mitosis correlating with an increase in its phosphorylation. A global mass spectrometry-based analysis of recombinant p12-chromatin interactions, identified significant overlap with the chromatin interactome of PFV CBS. Importantly, using biolayer interferometry, we were able to detect direct binding of several p12 orthologs, including WT Mo-MLV p12, to purified recombinant nucleosomes, and show that these p12 proteins could compete with PFV CBS for chromatin. This suggests a shared evolutionary mechanism for histone binding between gammaretroviruses and foamy viruses.

## Results

### Mutations in the MLV p12 CTD do not affect CA binding *in vitro* or in virions

We recently demonstrated direct binding of purified N-tropic MLV (N-MLV) p12_WT to recombinant CA by utilising an assay that was established for studying the interactions of CA with the restriction factor Fv1 [9, 26]. In this assay, His-tagged N-MLV CA was immobilised on lipid tubes, comprising Ni-chelating DGS-NTA, to enable it to adopt a regular hexameric arrangement. Although an NTD mutant of p12 did not interact with CA, we did not evaluate p12 CTD mutants for CA binding. Therefore, to investigate the contribution of the p12 CTD in the interaction with CA, we compared the binding of purified N-MLV p12_WT, p12_mut6 (an NTD mutant, Fig 1A) and p12_mut14 (a CTD mutant, Fig 1A) proteins to CA-coated lipid tubes. Purified p12 was incubated with CA-coated tubes and CA complexes were separated from unbound proteins by centrifugation through a sucrose cushion. The pellets were subsequently probed for p12 and CA by western blotting. In contrast with p12_mut6, p12_mut14 showed similar binding to WT CA as p12_WT (Fig 1B, lane 2). Furthermore, neither p12_mut14 nor p12_WT bound P1G CA which does not form regular arrays (Fig 1B, lane 3). These results therefore suggest that alterations in the CTD of p12 do not significantly affect CA binding.

Although purified p12 appears to bind recombinant CA arrays *in vitro*, an association between p12 and CA in viral particles has not yet been demonstrated. In fact, in a previous study, CA co-immunoprecipitated with p12 from lysates of Mo-MLV infected cells but not from lysates of extracellular virions [11]. To demonstrate an association between p12 and CA in virions, we next performed co-immunoprecipitation (Co-IP) assays using lysates from purified virion-like particles (VLPs). Prior to performing the immunoprecipitations, the viral lysates were normalised based on their CA content and treated with 1% formaldehyde. Due to its short cross-linking span (2–3 Å), formaldehyde is commonly used to facilitate the detection of specific protein-protein interactions [27]. Importantly, an antibody targeting p12 was able to immunoprecipitate CA in our assays (Fig 1C). In comparison with p12_WT (Fig 1C, lane 2), the amount of CA that co-immunoprecipitated was similar for p12_mut14 (Fig 1C, lane 6) but much lower for p12_mut6 (Fig 1C, lane 4).

The CA shell of the mature MLV virion is composed of a network of hexagonal rings [28]. To identify whether p12 recognises monomeric or hexameric CA, we next probed the binding of purified p12 to His-tagged CA immobilised on either lipid nanotubes or on Ni-NTA beads. Unlike on lipid nanotubes, the CA molecules arrayed on beads would be expected to be randomly-oriented. As shown in Fig 1D, even though the Ni-NTA beads carried similar amounts of CA as the lipid tubes, they did not detectably bind p12 (Fig 1D, lane 4). Our results therefore suggest that, similarly to CA-binding restriction factors [26], p12 only recognises hexameric CA. In addition, we used the lipid tube-based binding assay to determine the approximate stoichiometry of the p12-CA interaction (S1 Fig). For this purpose, the binding assays were performed with a 16-fold molar excess of p12 to promote the occupation of all potential p12 binding sites on CA. Comparing the immunoblot band intensities to those of a standard curve of known p12 protein concentrations, we calculated the amount of p12 pelleted by the CA-nanotubes as 11.17 pmol. Given that we loaded 67 pmol CA onto the nanotubes, this gives a p12:CA ratio of 1:6, which is suggestive of an interaction surface which is formed upon CA hexamerisation. However, this stoichiometry of one p12 molecule per CA hexamer may be an under-estimation as some CA molecules immobilised on the tubes may not be present in the exact hexameric arrangement conducive to p12 binding.

### The Mo-MLV p12 CTD tethers the CA-containing MLV PIC to host mitotic chromatin independently of IN-BET interactions

**Fig 2:**
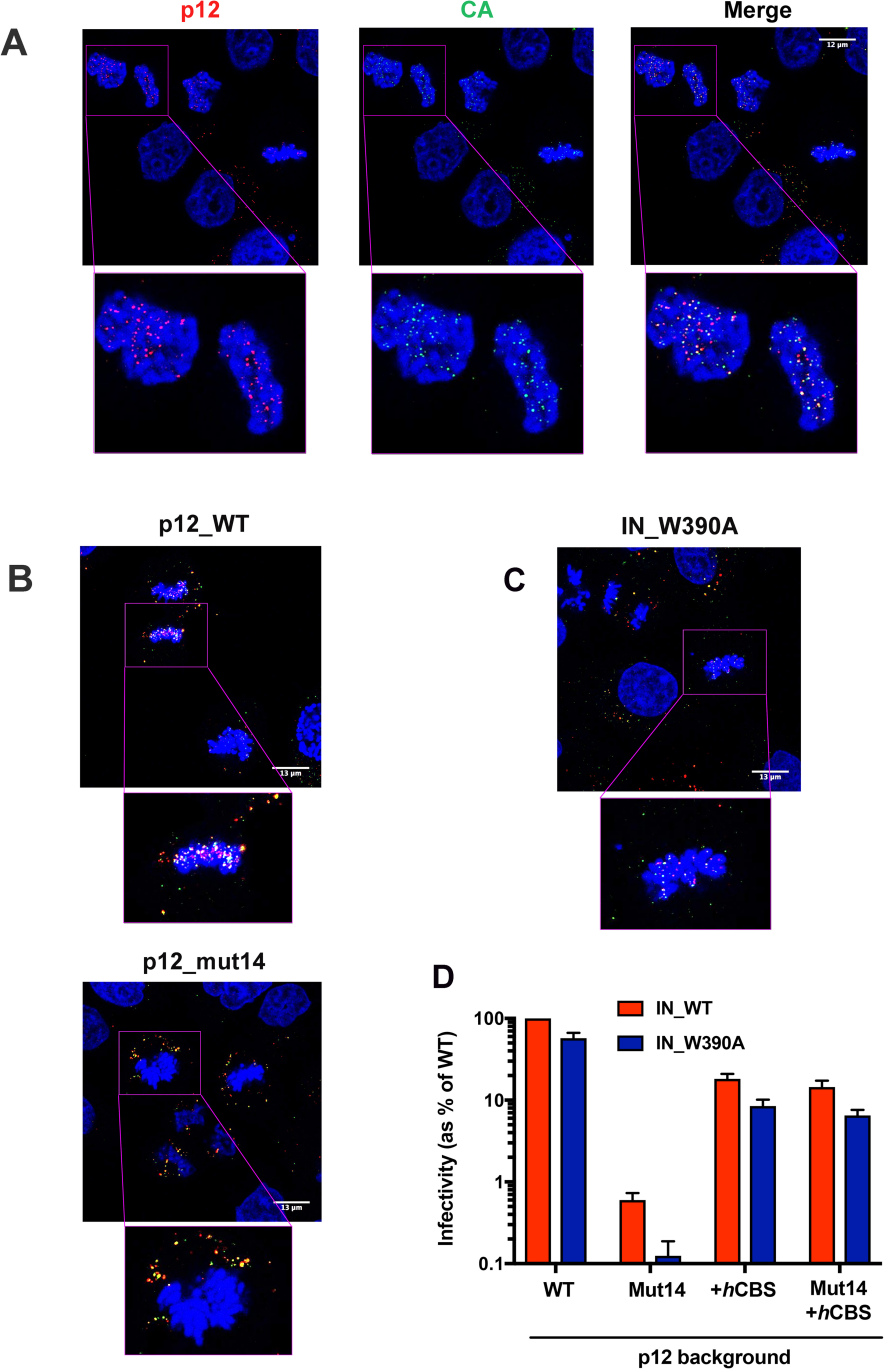
CA and p12 co-localise on mitotic chromatin independently of BET-protein binding. HeLa cells synchronised using a double-aphidicolin block were infected with WT Mo-MLV VLPs or mutants carrying p12_mut14 or IN_W390A. 10 h post-infection, the cells were fixed, stained for p12 (anti-p12, red), CA (anti-CA, green) and DNA (DAPI, blue), and analysed by confocal microscopy. (A) Representative images showing WT p12 and CA co-localisation on mitotic chromatin. Bottom panels are enlarged views of boxed regions in top panels. (B) Representative images of cells infected with VLPs carrying p12_WT (top panels) or p12_mut14 (bottom panels). (C) A representative image of cells infected with VLPs carrying IN_W390A, which is deficient in BET protein binding. (D) Infectivity of Mo-MLV VLPs carrying alterations in p12 and/or IN. HeLa cells were challenged with equivalent RT units of *LacZ*-encoding VLPs carrying p12_WT, p12_mut14, p12+*h*CBS or (p12+*h*CBS)_mut14 in combination with IN_WT or BET-binding deficient IN_W390A. Infectivity was measured 72 h post-infection by detection of beta-galactosidase activity in a chemiluminescent reporter assay. The data are plotted as percentage of WT VLP infectivity (mean ± SEM of >3 biological replicates).

As the CTD of p12 did not appear to interact with CA, we next asked how alterations in the p12 CTD affected CA localisation during virus replication. We therefore immunostained Mo-MLV-infected cells for p12 and CA simultaneously. Cells were synchronised by treatment with aphidicolin and then released from the G1/S block 30 minutes prior to infection. Cells were stained 10 h post-infection. Interestingly, in contrast to a previous study [11], CA was observed to co-localise with p12 on mitotic chromatin in cells infected with WT VLPs (Fig 2A). Quantitative analysis of chromatin-associated viral complexes in twelve mitotic cells (~45 tethered complexes/cell on average) revealed that 57±6% of p12-puncta were co-stained for CA and that 68±4% of CA-puncta were co-stained for p12. In mitotic cells infected with VLPs carrying p12_mut14, neither p12 nor CA tethered to chromatin (Fig 2B). However, p12 and CA still co-localised in the cytosol of these cells (39±4% of p12-puncta co-localised with CA and 54±5% of CA-puncta co-localised with p12 in five mitotic cells). Our results therefore suggest that binding of CA to p12 is not disrupted upon p12 CTD-mediated chromatin association, identifying how p12 can act as a tether between host chromatin and the viral PIC.

In addition to p12, MLV also carries another protein known to interact with chromatin, IN. In fact, target site selection for MLV integration is known to be mediated by the binding of IN to chromatin-associated BET proteins [20–22]. How the IN-BET interaction contributes to mitotic chromatin tethering of the MLV PIC is not known. We therefore infected cells with VLPs carrying a mutant IN that is deficient for BET-protein binding, IN_W390A [29]. WT p12 and CA puncta were still observed to co-localise with mitotic chromatin in these cells (Fig 2C), suggesting that the IN-BET interactions are not essential for chromatin tethering during mitosis. Furthermore, whereas VLPs carrying mutations in the CTD of p12 (p12_mut14) were severely defective in replication (>150-fold compared to WT VLPs), IN_W390A containing VLPs showed only a mild defect of <2-fold in our assays (Fig 2D). Our results therefore suggest that IN-BET interactions may not be as critical for MLV replication *per se* compared to p12-chromatin interactions. Interestingly, whereas the insertion of a heterologous chromatin binding sequence (*h*CBS) from the prototypic foamy virus (PFV) into p12 increased the replication of p12_mut14 carrying VLPs, it did not rescue the slight infectivity defect of the IN_W390A mutation (Fig 2D). Thus, p12- and IN-chromatin interactions likely have distinct functions for the virus.

### Recombinant Mo-MLV p12 does not show detectable association with mitotic chromatin

**Fig 3:**
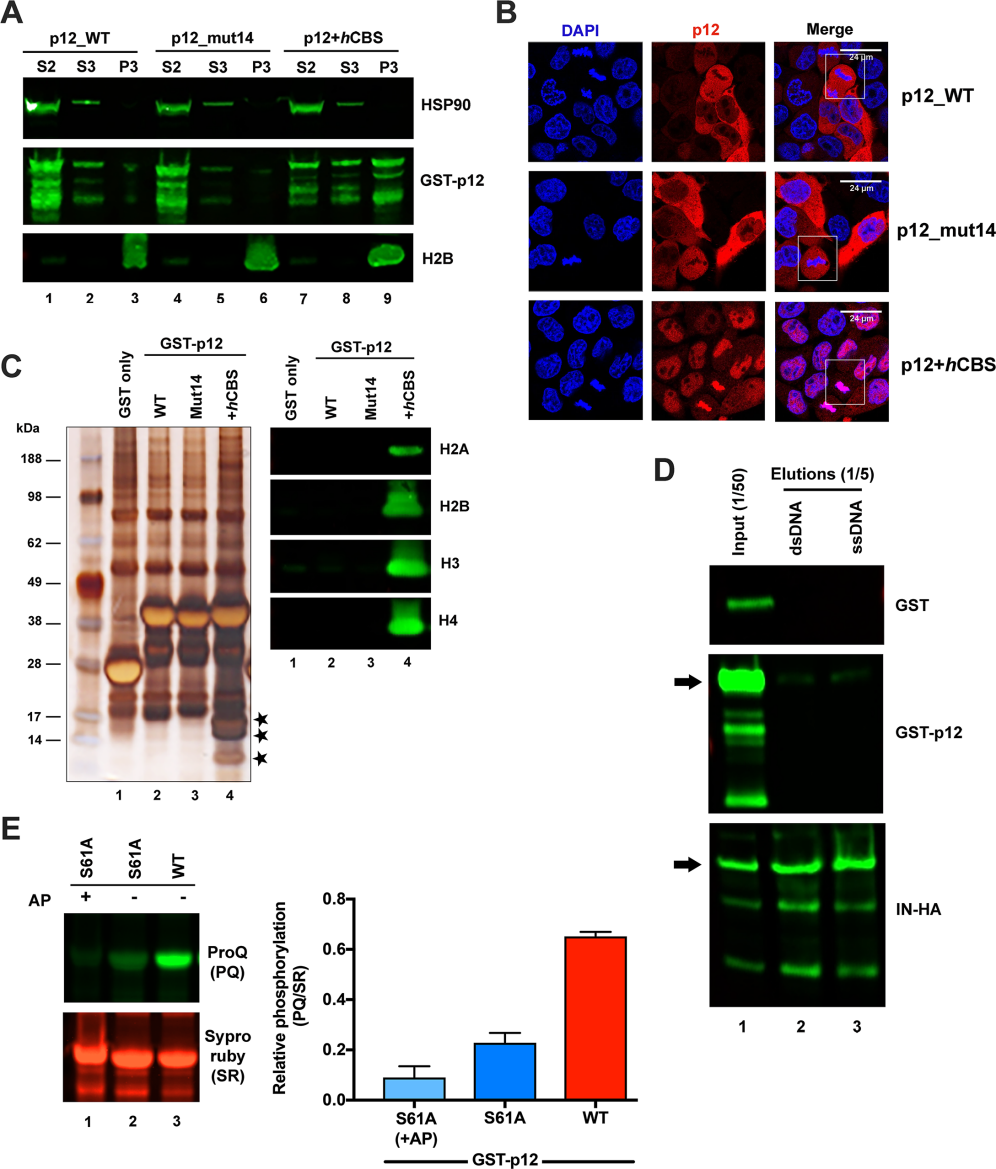
Recombinant GST-Mo-MLV p12 does not associate with mitotic chromatin but is phosphorylated. (A) A representative immunoblot showing subcellular distribution of GST-p12. GST-tagged Mo-MLV p12_WT (lanes 1–3), p12_mut14 (lanes 4–6) and p12+*h*CBS (lanes 7–9) were expressed in 293T cells for ~40 h. Cells were then subjected to biochemical fractionation and equivalent amounts of fractions S2-cytosolic (lanes 1, 4 and 7), S3-soluble nuclear (lanes 2, 5 and 8) and P3-chromatin pellet (lanes 3, 6 and 9) were analysed by SDS-PAGE and immunoblotting with anti-p12, anti-HSP90 (cytosolic marker) and anti-H2B (chromatin marker) antibodies. (B) Representative confocal microscopy images showing GST-p12 localisation in HeLa cells stably transduced with constructs expressing GST-tagged Mo-MLV p12_WT, p12_mut14 or p12+*h*CBS. Cells were stained for p12 (anti-p12, red) and DNA (DAPI, blue). White boxes indicate mitotic cells. (C) Representative silver-stained SDS-PAGE gel (left) and immunoblot (right) of GST-p12 complexes. 293T cells were transiently-transfected with expression constructs for GST-tagged Mo-MLV p12_WT (lane 2), p12_mut14 (lane 3) or p12+*h*CBS (lane 4), or GST alone (lane 1). 24 h post-transfection, cells were treated with nocodazole overnight to arrest them in mitosis and then lysed. Cell lysates were normalised on total protein concentration and GST-p12 protein complexes were precipitated with glutathione-sepharose beads. Bead eluates were analysed by SDS-PAGE followed by silver-staining or immunoblotting with anti-H2A, anti-H2B, anti-H3 or anti-H4 antibodies. Bands corresponding to core histones in the silver-stained gel are starred. (D) Immunoblot showing DNA pull down assays. 293T cells were transiently-transfected with expression constructs for GST alone (top panel), GST-tagged Mo-MLV p12_WT (middle panel), or IN-HA (bottom panel) for ~40 h. DNA interacting proteins were precipitated from normalised cell lysates with cellulose beads coated with double stranded (lane 2) or single-stranded (lane 3) calf thymus DNA, and analysed by immunoblotting with anti-GST, anti-p12, or anti-IN antibodies, respectively. The arrows indicate full-length GST-p12 (~38 kDa) and IN-HA (~49 kDa) bands in the western blots. (E) GST-p12 phosphorylation. Normalised, mitotic cell lysates expressing GST-tagged Mo-MLV p12_WT (lane 3) or p12_S61A (lanes 1 and 2) were incubated with glutathione-sepharose beads. Bound proteins were separated by SDS-PAGE and the gel was sequentially stained with ProQ diamond (PQ, specifically stains phosphorylated proteins) and Sypro ruby (SR, stains all proteins) dyes. Prior to SDS-PAGE, one p12_S61A sample was treated with alkaline phosphatase (AP) for 1 h at 37°C. Band intensities were measured using a ChemiDoc imaging system and the bar chart shows PQ/SR ratios, plotted as mean ± SD of 3 technical replicates.

Having shown that the chromatin localisation of CA-containing PICs in infected cells was dependent on the p12 CTD, we next wanted to investigate the interactions of the p12 CTD with cellular proteins. We therefore synthesised N-terminally GST-tagged Mo-MLV p12 and analysed its expression and sub-cellular localisation. Biochemical fractionation of cycling 293T cells transiently expressing GST-p12 revealed that, surprisingly, recombinant WT p12 was mainly cytoplasmic, being detected in the same fraction as HSP90, a cytosolic marker, and not in the fraction containing histone H2B, a chromatin marker (Fig 3A, lanes 1–3). Similar localisation was observed with GST-tagged p12_mut 14 (Fig 3A, lanes 4–6). The PFV *h*CBS is known to bind histones H2A-H2B [14, 15] and GST-p12 carrying this motif (p12+*h*CBS) could be readily detected in the chromatin pellet fraction (Fig 3A, lane 9), suggesting that the GST-tag itself is unlikely to be sterically interfering with chromatin tethering. In general, mitotic cells comprise only a small fraction of a cycling cell population. Therefore, to distinguish between p12 localisation in interphase and mitotic cells, we probed the sub-cellular distribution of p12 by immunostaining HeLa cells stably-expressing GST-p12 (Fig 3B). In agreement with the fractionation assays, stably-expressed p12+*h*CBS was enriched in the nuclei of interphase cells and localised on the chromatin of mitotic cells (Fig 3B). However, both p12_WT and p12_mut14 were mainly cytosolic in interphase cells and did not show a detectable association with mitotic chromatin in the few mitotic cells present (Fig 3B).

To enrich cells in mitosis and look directly for an interaction with chromatin, we arrested 293T cells transiently expressing GST-p12 (~38 kDa) in metaphase, by nocodazole treatment, prior to their lysis for pull-down assays. First, cell lysates normalised for total protein concentration were incubated with glutathione-sepharose beads. The eluates from the beads were subsequently analysed by SDS-PAGE followed by silver-staining (Fig 3C, left panel) and immunoblotting (Fig 3C, right panel). The core histones, H2A, H2B, H3 and H4, were all pulled-down with p12+*h*CBS in our assays (Fig 3C, lane 4) but not with p12_WT (Fig 3C, lane 2). These GST pull-down assays were performed with lysates pre-treated with nuclease to eliminate the detection of DNA- or RNA-mediated protein interactions. Therefore, to determine whether GST-p12 was able to bind DNA we also incubated mitotic lysates of transfected cells with DNA-coated cellulose beads. HA-tagged IN, used as a positive control, showed binding to beads coated with both single- and double-stranded DNA (Fig 3D, bottom panel). However, no binding to DNA was observed with GST-p12 (Fig 3D, middle panel). Some additional minor bands were seen on both GST-p12 and IN-HA blots, which were likely break down products.

Overall, four separate assays suggested that GST-p12 is unable to bind chromatin when expressed recombinantly. This concurs with a previous study, in which a Mo-MLV p12 fusion protein carrying a C-terminal GFP tag did not show detectable association with mitotic chromatin by fluorescence microscopy [19]. In that study, the inability of p12-GFP to associate with chromatin was attributed to a lack of phosphorylation of recombinant p12 [19]. To test this hypothesis in our system, we determined the phosphorylation status of GST-p12 in mitotic cells.

### Recombinant Mo-MLV p12 is phosphorylated

Firstly, GST-p12 pulled-down from mitotic cell lysates was analysed by SDS-PAGE followed by sequential staining with two fluorescent dyes, ProQ diamond (PQ) and Sypro Ruby (SR) [30, 31]. PQ is a small molecule fluorophore that binds with high-specificity to phosphorylated proteins. SR binds all proteins and is useful as a total protein indicator. The ratio of PQ and SR dye signals provides a measure of the phosphorylation level with respect to the total amount protein. Viral p12 is known to be mainly phosphorylated at serine 61 within the CTD [19, 25]. We therefore compared the relative phosphorylation levels of GST-p12_WT with a S61A mutant. Compared to p12_S61A, p12_WT was at least 3-fold more phosphorylated (Fig 3E, lanes 2 and 3). Treatment of p12_S61A with alkaline phosphatase further reduced the phosphorylation level by ~2-fold (Fig 3E, lanes 1 and 2), suggesting that recombinant p12 is also phosphorylated at sites other than S61.

We next used nanoscale liquid chromatography coupled to tandem mass spectrometry (nano LC–MS/MS) to identify phosphopeptides of GST-p12_WT precipitated from 293T cells. The phosphorylation sites in the high confidence tryptic-peptides (<1% false discovery rate, FDR) were assigned using the probability-based phosphoRS algorithm [32] (Table 1). A peptide was counted as being phosphorylated at a specific residue if its phosphoRS site probability was >50%. For each tryptic peptide, the phosphorylation level at a particular site was estimated by dividing the phosphorylated peptide count by the total peptide count. This analysis, carried out with two biological replicates, confirmed S61 to be the main site of phosphorylation on GST-p12_WT, with ~45% of S61-containing peptides phosphorylated at this residue (Table 1). We also identified T52, S65, S78, T80 and S81 as additional sites of low-level phosphorylation on p12 (Table 1). Our results suggest that, like viral p12, recombinant GST-p12 is phosphorylated at S61. The absence of a detectable association between GST-p12 and mitotic chromatin is therefore unlikely to result from a lack of phosphorylation of recombinant p12.

**Table 1:** Identifying phosphorylation sites of GST-p12 precipitated from 293T cells using nano LC–MS/MS.

Phosphorylated peptides	Missed cleavages	^1^Phosphorylated residues (>50% probability)	^2^Fraction of phosphorylated peptides	
			^3^R1	R2
DGNGGEATPAGEAPDPSPMASR (p12 aa 45–66)	0	T52	0.13	0.04
		**S61**	**0.36**	**0.49**
		**S65**	**0.01**	**0.07**
DPRPPPSDRDGNGGEATPAGEAPDPSPMASR (p12 aa 36–66)	1	T52	ND	ND
		**S61**	**0.47**	**0.52**
		**S65**	**ND**	**ND**
EPPVADSTTSQAFEFGGR (p12 aa 72–84+ linker)	0	S78	0.03	0.03
		T80	0.01	ND
		S81	0.03	0.01
REPPVADSTTSQAFEFGGR (p12 aa 71–84+ linker)	1	S78	ND	ND
		T80	0.04	ND
		S81	0.10	ND

^1^ A peptide was counted as being phosphorylated at a specific residue if its *phosphoRS* site probability was >50%.

^2^ For each tryptic peptide, the phosphorylation level at a particular site was estimated by dividing the phosphorylated peptide count by the total peptide count.

^3^ R1 and R2 are biological replicates.

### Recombinant Mo-MLV p12 recapitulates the known interactions of Gag p12

The inability of recombinant Mo-MLV p12 to bind mitotic chromatin even when phosphorylated suggests that the affinity of p12 for chromatin may be influenced by other viral factors. We have observed viral p12 to be bound to CA when it associates with mitotic chromatin in Mo-MLV infected cells (Fig 2A). Prior to Gag cleavage, p12 is thought to be in a largely unstructured conformation [33]. In mature virions, the interaction of the p12 NTD with the CA lattice may induce a conformational change that increases the affinity of the p12 CTD for chromatin. In fact, such a conformational switch could potentially be important in the temporal regulation of late and early life cycle events of p12. As part of Gag, p12 is known to interact with homologous to E6AP COOH terminus (HECT) ubiquitin ligase, WWP2, via the Late (L)-domain motif, and with clathrin, CLTC, via the N-terminal DLL motif [5, 34]. As these interactions do not require p12 to be bound to CA, they should, in theory, be recapitulated by recombinant GST-p12 in our system.

**Fig 4:**
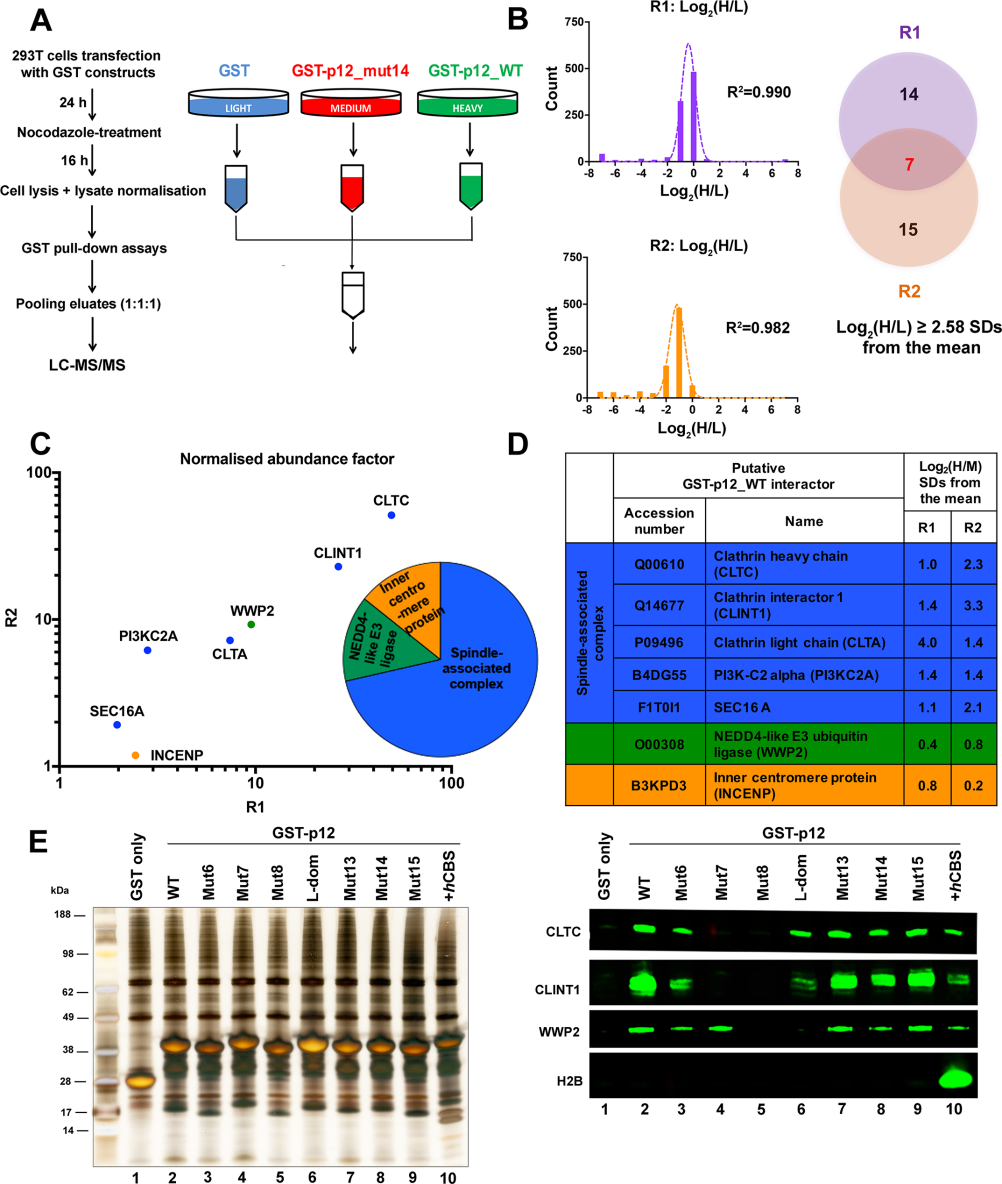
GST-Mo-MLV p12 recapitulates known interactions of the p12 region of Gag. Cellular proteins interacting with GST-p12 were identified using SILAC-MS. Two biological repeats (R1 and R2) were performed. (A) Schematic diagram of the SILAC-MS workflow. GST-protein complexes were isolated from normalised mitotic 293T cell lysates using glutathione-sepharose beads, pooled and subjected to LC-MS/MS analysis. (B) Identification of proteins enriched in the heavy-labelled GST-p12_WT (H) sample relative to light-labelled GST (L) sample. Log_2_(H/L) silac ratios of the set of MS hits (FDR <5%) from each replicate (R1 and R2) were plotted as a frequency distribution. Mean and standard deviation (SD) of each distribution was estimated by fitting to a normal distribution curve (R^2^ ≥ 0.98). MS hits with log_2_(H/L) ratios greater than 2.58 SDs from the mean were selected as significantly enriched in the GST-p12_WT (H) sample. Of the 21 such proteins identified in R1, 7 were also found in R2 (Venn diagram), and were investigated further. (C) Ranking of the 7 potential GST-p12_WT binding proteins. The abundance factor for each protein was calculated by dividing the peptide spectral count by its length. These values were then normalised across the group and the scores for R1 plotted against the scores for R2. The points are coloured according to the protein function shown in the pie chart. CLTC = clathrin heavy chain, CLINT1 = clathrin interactor 1, WWP2 = NEDD4-like E3 ubiquitin ligase, CLTA = clathrin light chain, PI3KC2A = phosphatidylinositol-4-phosphate-3-kinase C2, SEC16A = protein transport protein Sec16A, INCENP = inner centromere protein. (D) The relative enrichment of the 7 proteins (putative GST-p12_WT interactors) in the GST-p12_WT (H) samples compared to GST-p12_mut14 (M) samples was estimated from the log_2_(H/M) ratios. (E) Representative silver stained gel (left) and immunoblot (right) showing binding of a panel of GST-p12 mutants to selected host proteins. Mutant nomenclature is shown in Fig 1. 293T cells were transiently-transfected with expression constructs for GST-tagged Mo-MLV p12_WT (lane 2), p12 mutants (lanes 3–10) or GST (lane 1) for ~24 h before being treated overnight with nocodazole. GST-p12 protein complexes were precipitated from normalised cell lysates with glutathione-sepharose beads and analysed by SDS-PAGE followed by silver-staining or immunoblotting with anti-CLTC, anti-CLINT1, anti-WWP2 and anti-H2B antibodies.

To test whether GST-p12 was acting like the p12 region of Gag, we used a global proteomic approach based on stable isotope labelling by amino acids in cell culture (SILAC)-mass spectrometry (MS) to look for host proteins that interact with GST-p12 [35, 36]. A schematic diagram of the workflow is illustrated in Fig 4A. Briefly, GST, GST-p12_mut14 and GST-p12_WT were transiently-expressed in 293T cells cultured in light (R0/K0), medium (R6/K4) and heavy (R10/K8) SILAC media, respectively. Next, lysates of nocodazole treated transfected cells were prepared, normalised and used for parallel pull-down assays with glutathione-sepharose beads. The eluates from the beads were then pooled in a 1:1:1 ratio and subjected to LC-MS/MS analysis. The experiment was performed using biological replicates to test reproducibility of the mass-spec hits. The pull-down assays for this experiment were performed using mitotic cell lysates to facilitate identification of any potential chromatin interactions that were previously missed.

After removal of obvious contaminants (e.g. keratin) and non-quantified hits, 948 and 879 proteins were identified in replicates 1 and 2 (R1 and R2) of the experiment respectively (the false discovery rate (FDR) was set at 5%). To select proteins enriched in the heavy-labelled (H) GST-p12_WT sample relative to the light-labelled (L) GST sample, we compared the log_2_(H/L) silac ratios of the mass-spectrometry hits. When plotted as a frequency distribution, the log_2_(H/L) ratios of each replicate fitted well to a normal distribution curve (R^2^ ≥ 0.98), allowing the mean and standard deviation to be accurately estimated (Fig 4B). In this experiment, contaminants/non-specifically bound cellular proteins were assumed to cluster around the mean of the distribution. Therefore, hits with log_2_(H/L) ratios greater than 2.58 standard deviations (SDs) from the mean were considered to be significantly enriched (99% confidence threshold, *p*≤0.01) in the GST-p12_WT sample. Twenty one such proteins were identified in R1 and 22 identified in R2 (Fig 4B Venn diagram). Of these, seven proteins were found in both R1 and R2, suggesting that they may bind recombinant p12_WT (Fig 4B and S1 Table). These proteins were then ranked based on normalised abundance in the group. In mass-spectrometry analysis, larger proteins are in general assigned higher scores. Therefore, to control for protein size, we divided the number of peptide spectral matches identified for each protein with its length and then normalised the values across the group (Fig 4C and 4D). When ranked this way, the clathrin heavy chain (CLTC), clathrin interactor 1 (CLINT1) and NEDD4-like E3 ubiquitin ligase (WWP2) were identified as the top three hits. As CLTC and WWP2 are known interactors of the p12 region of Gag, our results suggest that recombinant p12 may be more representative of immature p12. The function of clathrin in MLV infection is unclear. Interestingly, in mitosis, CLTC has recently been found to play a role in cross-linking the kinetochore microtubules of the spindle [37]. Furthermore, of the seven proteins identified as p12_WT binders in this experiment, four (including CLINT1) have previously been found to associate with CLTC in a mitotic spindle stabilising complex [38]. Thus, clathrin binding may be relevant to the early stages of MLV replication and should be investigated further.

To validate these mass-spectrometry results, we next performed glutathione-sepharose pull-down assays with mitotic lysates containing GST-tagged WT p12 and a panel of p12 mutants (the mutations are annotated in Fig 1A). The eluates from the assays were subsequently analysed by silver-staining (Fig 4E, left panel) and immunoblotting (Fig 4E, right panel). CLTC and CLINT1 were pulled-down with all p12 proteins apart from the NTD mutants 7 and 8 (Fig 4E, lanes 4 and 5). This is consistent with the location of the CLTC-binding motif, DLL in p12: In p12_mut8, the DLL motif is substituted with alanines. In p12_mut7, residues neighbouring the DLL motif are mutated which may also inhibit CLTC recruitment. The interaction between p12 and WWP2 was also re-capitulated as expected in the assays, with WWP2 being pulled-down with all p12 proteins apart from the L-domain mutant and p12_mut8.

To identify potential chromatin interactions of p12, we compared the heavy-labelled (H) GST-p12_WT sample to the medium-labelled (M) GST-p12_mut14 sample, as viruses carrying p12_mut14 do not localise to chromatin (Fig 2B). However, after performing a similar analysis with the log_2_(H/M) silac ratios of the mass-spectrometry hits as above (S2 Fig), we found no proteins that passed the criteria for significant enrichment (log_2_(H/M) ratio > 2.58 SDs from the mean) in both replicates (Fig 4D and S2 Fig). The lack of a significant difference in the interactions of recombinant p12_WT and p12_mut14 suggests that the CTD functions of viral p12 are not replicated by GST-p12 in this system.

Based on the observations above, recombinant GST-p12 appears to mimic Gag-p12 as it was able to recapitulate the known interactions of the p12 region of Gag, which are mediated by motifs within or next to the NTD region of p12. However, GST-p12 did not show detectable chromatin interactions, unlike viral p12 in the PIC. We therefore suggest that another viral protein, probably CA, influences the ability of p12 to interact with chromatin.

### A single amino acid change in recombinant Mo-MLV p12 can confer an interaction with mitotic chromatin

There are a number of potential mechanisms by which CA may facilitate the interaction of the p12 CTD with mitotic chromatin. If the p12 NTD sterically-hinders the CTD from interacting with chromatin, the binding of CA to the NTD could remove this inhibition. On the other hand, CA binding may prevent host proteins, like clathrin, from binding to p12 and sterically hindering a chromatin interaction. Alternatively, binding to CA may induce a subtle but important conformational change in the peptide backbone of p12 which increases its affinity for chromatin. To potentially distinguish between these possible mechanisms, we introduced a range of alterations into Mo-MLV GST-p12 and tested the mutants for chromatin interactions. In the p12_CTD only mutant, the p12 NTD was deleted to remove any steric hindrance from this domain. In the p12_D25A/L-dom mutant, the CLTC and WWP2 binding sites were removed by substituting alanine at D25 of the DLL motif and at the PPPY L-domain motif, respectively. The other two mutants included in the panel, p12_M63I and p12_G49R/E50K, carried changes that were previously identified in an *in vitro* evolution study to find viral revertants that compensated for the non-infectious Mo-MLV p12_SS(61,65)AA mutant that lacks phosphorylation [25]. Recently the M63I substitution has also been shown to rescue chromatin tethering of recombinant Mo-MLV p12-GFP [19].

**Fig 5:**
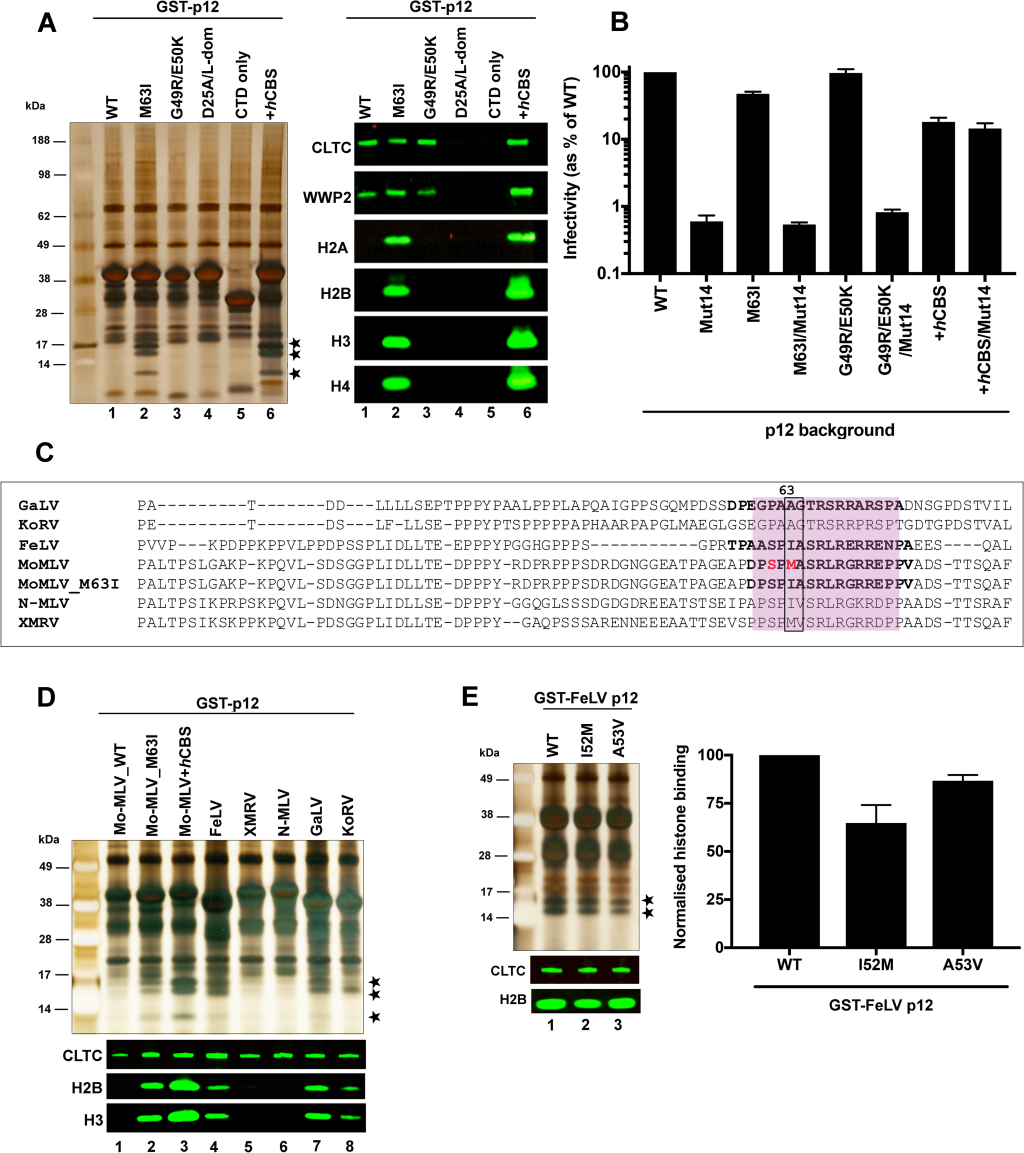
GST-Mo-MLV p12_M63I and other p12 orthologs associate with mitotic chromatin. (A) Representative silver stained gel (left) and immunoblot (right) showing binding of a panel of GST-p12 mutants to host proteins. 293T cells were transiently-transfected with expression constructs for GST-tagged Mo-MLV p12_WT (lane 1) and a panel of Mo-MLV p12 mutants: M63I (lane 2), G49R/E50K (lane 3), D25A/L-dom (carrying alanine substitutions of the PPPY motif as well as D25A, which disrupts clathrin binding, lane 4), p12 CTD only (lane 5) or GST-p12+*h*CBS (positive control, lane 6) for ~24 h before being treated overnight with nocodazole. GST-p12 protein complexes were precipitated from normalised cell lysates with glutathione-sepharose beads and analysed by SDS-PAGE followed by silver-staining or immunoblotting with anti-CLTC, anti-WWP2, anti-H2A, anti-H2B, anti-H3 and anti-H4 antibodies. Bands corresponding to core histones in the silver-stained gel are starred. (B) Infectivity of Mo-MLV VLPs carrying alterations in p12. HeLa cells were challenged with equivalent RT units of *LacZ*-encoding VLPs carrying Mo-MLV p12_WT, M63I, G49R/E50K or p12+*h*CBS +/- Mut14, and infectivity was measured 72 h post-infection by detection of beta-galactosidase activity in a chemiluminescent reporter assay. The data are plotted as percentage of WT VLP infectivity (mean ± SEM of >3 biological replicates). (C) An alignment of p12 sequences from selected gammaretroviruses. The CTD region is shaded pink. The S61 and M63 residues of Mo-MLV p12 are highlighted in red and equivalent residues at position 63 and 64 are boxed. CTD peptide sequences used in subsequent BLI assays (Fig 9) are in bold. (D and E) Representative silver stained gel (top) and immunoblot (bottom) showing interaction of a panel of GST-tagged p12 orthologues (D) and GST-tagged FeLV_p12 mutants I52M and A53V (E) to chromatin associated proteins. GST-pull down assays were performed as in (A). (E) The amount of histone H2B pulled-down with GST-p12 was quantified for each sample by estimating median band intensity of immunoblots using a Li-cor Odyssey imaging system and plotted in the bar chart as mean ± SD of 3 technical replicates.

We first tested the panel of p12 mutants in pull-down assays from mitotic cells using glutathione-sepharose beads (Fig 5A). GST-tagged p12_WT and p12+*h*CBS were included in these assays as negative and positive controls for chromatin binding, respectively. Analysis of bead eluates by silver-staining and western blotting revealed that core histones were pulled-down with p12_M63I (Fig 5A, lane 2) but not with the other mutants (Fig 5A, lanes 3–5). Probing the sub-cellular distribution of the mutants by immuno-staining of stably-transduced HeLa cell lines (S3 Fig) only detected a chromatin association of p12_M63I and p12+*h*CBS during mitosis. In agreement with the immunoprecipitation data, the other mutants were excluded from mitotic chromatin. Thus, removing the p12 NTD or preventing known interactions with host proteins at/near the NTD was unable to confer chromatin binding to GST-p12. Instead, this was attained by the M63I substitution in the p12 CTD. Surprisingly, the G49R/E50K mutation, which also rescues the infectivity of the SS(61,65)AA mutant, was unable to confer chromatin tethering of GST-p12 in our assays. M63I, is located within the p12 CTD, whereas the G49R/E50K changes are located outside of the CTD region. Therefore, perhaps only the M63I change alters the conformation of the chromatin binding region of p12 directly. Overall, our results suggest that the inability to detect an interaction between phosphorylated GST-p12_WT and chromatin in the absence of CA is probably not due to steric inhibition by the p12 NTD or cellular proteins but due to the p12 CTD being in a conformation that is not conducive for detectable binding.

We also tested the M63I and G49R/E50K mutants in infectivity assays (Fig 5B). VLPs carrying p12_G49R/E50K showed similar infectivity to WT Mo-MLV, whereas p12_M63I VLPs had a mild ~2-fold defect. As p12+*h*CBS VLPs also show a moderate defect (~5-fold) in infectivity compared to WT Mo-MLV, our results corroborate the hypothesis that increasing the affinity of Mo-MLV p12 for chromatin, above WT levels, may be detrimental to viral replication [12]. Furthermore, the M63I and G49R/E50K mutations were unable to rescue the infectivity of p12_mut14 VLPs (Fig 5B). This implies that the M63I mutation modulates an existing p12 interaction rather than providing an independent interaction like the PFV *h*CBS.

### Recombinant p12 proteins from other gammaretroviruses interact with mitotic chromatin

It was somewhat surprising that the conservative single amino acid change of methionine-63 to isoleucine could have a dramatic effect on the chromatin interaction of p12. Interestingly, we noted that the p12 orthologues from N-tropic (N)-MLV and feline leukaemia virus (FeLV) naturally had an isoleucine residue at position 63 (Fig 5C). We have previously shown that other gammaretroviral p12 proteins have similar functions to Mo-MLV p12 [7] and so wondered whether these orthologues may interact with chromatin when expressed recombinantly. We therefore expressed p12 from different gammaretroviruses as GST fusion proteins and tested them in pull-down assays (Fig 5D). Excitingly, core histones were pulled-down with p12 proteins from FeLV, gibbon ape leukaemia virus (GaLV) and koala retrovirus (KoRV) (Fig 5D, lanes 4, 7 and 8), but not with p12 from xenotropic MLV-related virus (XMRV) or N-MLV (Fig 5D, lanes 5 and 6). Recombinant gammaretroviral p12 proteins therefore appear to differ in their interactions with chromatin, with some p12-chromatin interactions being detectable in pull-down assays in the absence of CA and other viral proteins. The p12 CTD regions of FeLV, N-MLV and XMRV are >68% identical to Mo-MLV in sequence. The amino acids at residues equivalent to 63/64 in the different viruses are: M/A in Mo-MLV, I/A in FeLV, I/V in N-MLV and M/V in XMRV. As changing M63 to I, making it I/A, enhanced the chromatin interaction of Mo-MLV p12, and FeLV also has I/A at these positions and interacts with chromatin, we decided to investigate these residues further. We tested the effects of I52M and A53V mutants of FeLV p12 in pull-down assays. However, both I52M and A53V showed similar levels of histone precipitation (<2-fold difference) as the WT protein (Fig 5E), implying that the extent to which the I/A motif influences chromatin binding may be dependent on its sequence-context. Indeed, GaLV and KoRV p12 proteins which show only ~27% identity to Mo-MLV p12 in the CTD appear to interact with chromatin with sufficient affinity for *in vitro* detection in the absence of this motif.

### Chromatin interaction and phosphorylation of recombinant Mo-MLV p12 M63I is cell cycle-dependent

As the mode of chromatin binding appears to be conserved between Mo-MLV p12_WT and p12_M63I, both being dependent on residues 65–69 (Mut14) in p12, we used recombinant p12_M63I, which showed an association with mitotic chromatin in our biochemical assays, to characterise this interaction further.

In virus-infected cells, Mo-MLV p12 has only been observed to associate with chromatin between nuclear envelope disassembly and chromatin decondensation (Fig 2A) [11]. However, whether this reflects a preferential affinity of p12 for mitotic chromatin or merely the accessibility of chromatin during infection is unclear. Pre-mitosis, the MLV PIC is unable to actively traverse through the nuclear pores and post-mitosis, the dissociation of p12 from chromatin could be driven by other viral factors in the PIC. To investigate the cell-cycle dependency of the p12-chromatin interaction directly, we compared the chromatin interaction of recombinant Mo-MLV p12_M63I in interphase and mitotic cells.

**Fig 6:**
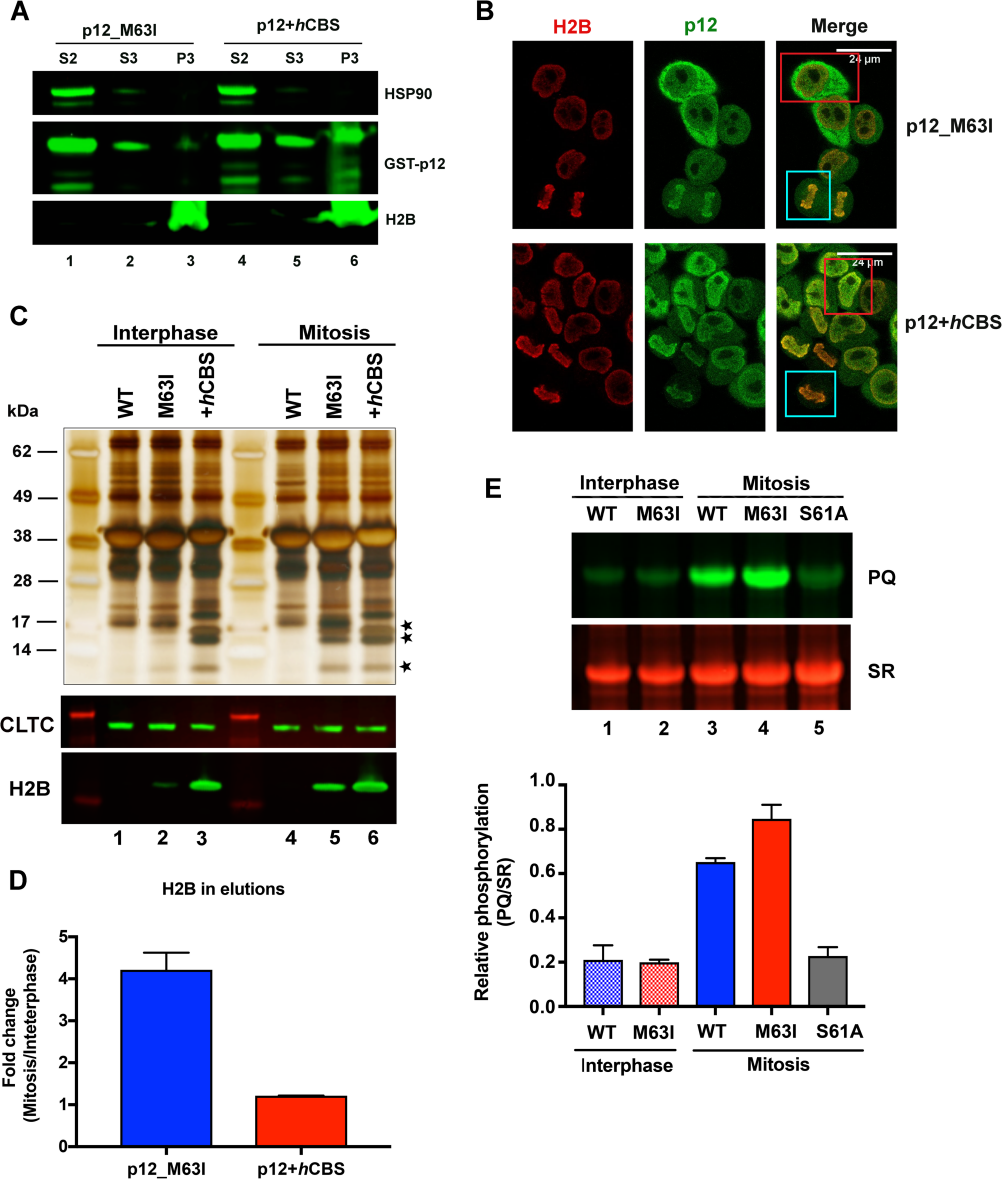
GST-tagged Mo-MLV p12_M63I shows increased chromatin association and phosphorylation in mitosis. (A) A representative immunoblot showing subcellular distribution of GST-p12 mutants. GST-tagged GST-p12_M63I (lanes 1–3) or GST-p12+*h*CBS (lanes 4–6) were expressed in 293T cells for ~40 h. Cells were then subjected to biochemical fractionation and equivalent amounts of fractions S2-cytosolic, S3-soluble nuclear and P3-chromatin pellet were analysed by SDS-PAGE and immunoblotting with anti-p12, anti-HSP90 (cytosolic marker) and anti-H2B (chromatin marker) antibodies. (B) Representative confocal microscopy images showing GST-p12 localisation in HeLa cells stably transduced with constructs expressing GST-p12_M63I and GST-p12+*h*CBS. Cells were stained for p12 (anti-p12, green) and H2B (anti-H2B, red). Blue boxes indicate mitotic cells and red boxes show interphase cells. (C) Representative silver stained gel (top) and immunoblot (bottom) comparing the interaction of GST-p12_M63I and GST-p12+*h*CBS with mitotic and interphase chromatin. 293T cells were transiently-transfected with expression constructs for GST-tagged Mo-MLV p12_WT, M63I or GST-p12+*h*CBS for ~24 h before being treated overnight with either nocodazole (to arrest in mitosis) or aphidicolin (to block in interphase). GST-p12 protein complexes were precipitated from normalised cell lysates with glutathione-sepharose beads and analysed by SDS-PAGE followed by silver-staining or immunoblotting with anti-CLTC and anti-H2B antibodies. Bands corresponding to core histones in the silver-stained gel are starred. (D) Quantitation of H2B pulled-down with GST-p12 from mitotic *versus* interphase cell lysates. Median H2B band intensities from immunoblots in (C) were measured using a Li-cor Odyssey imaging system. The increase in H2B precipitation from mitotic cell lysates relative to interphase cell lysates are plotted in the bar chart (mean ± SEM, three biological replicates). (E) GST-p12 phosphorylation in mitosis and interphase. Normalised, interphase or mitotic 293T cell lysates expressing GST-tagged Mo-MLV p12_WT, M63I or S61A were incubated with glutathione-sepharose beads. Bound proteins were separated by SDS-PAGE and the gel was sequentially stained with ProQ diamond (PQ, specifically stains phosphorylated proteins) and Sypro ruby (SR, stains all proteins) dyes. Band intensities were measured using a ChemiDoc imaging system and the bar chart shows PQ/SR ratios, plotted as mean ± SD of 3 technical replicates.

We first analysed the sub-cellular localisation of transiently-expressed GST-p12_M63I in cycling 293T cells using biochemical fractionation. When probed by immunoblotting, very little p12_M63I was detected in the chromatin pellet fraction (Fig 6A, lane 3) compared to the p12+*h*CBS control (Fig 6A, lane 6). To distinguish between p12_M63I localisation in interphase and mitotic cells, we next investigated the sub-cellular distribution of stably-expressed GST-p12_M63I by immunofluorescence. Whereas, GST-p12+*h*CBS was enriched on chromatin in both interphase and mitotic HeLa cells (Fig 6B, bottom panels, red and blue box, respectively), GST-p12_M63I showed a differential localisation (Fig 6B, top panels). It was chromatin-associated in mitosis (blue box), but mainly cytosolic in interphase cells (red box). The difference in the distribution of GST-p12_M63I and GST-p12+*h*CBS in interphase is likely due to differences in chromatin affinity rather than nuclear access. The *h*CBS sequence does not include a nuclear localisation signal [39] and as both GST_p12_M63I and GST_p12+*h*CBS are of similar size, they would be expected to progress at similar rates across the nuclear envelope by passive diffusion.

As GST-p12_M63I appeared to have a higher affinity for chromatin in mitosis compared to interphase, we performed glutathione-sepharose bead pull-down assays from cells arrested primarily in mitosis (>80% of cells in G2/M) or interphase (<2% of cells in G2/M) following treatment with nocodazole or aphidicolin, respectively. The final eluates from the beads were analysed by silver-staining and immunoblotting (Fig 6C, top and bottom panels, respectively). Approximately 4-fold more histones were pulled down with GST-p12_M63I from mitotic cell lysates than interphase cell lysates (Fig 6C, lanes 2 vs 5, Fig 6D), whereas GST-p12+*h*CBS showed similar levels of histone precipitation from both interphase and mitotic cell lysates (Fig 6C, lanes 3 vs 6, Fig 6D). The higher affinity of GST-p12_M63I for chromatin in mitosis could be due to changes in the post-translational state of p12 itself, increased expression of specific interacting proteins or changes in the putative chromatin target during this stage of the cell cycle. As phosphorylation of many proteins is cell-cycle dependent, we investigated whether the phosphorylation state of p12 changed in mitosis. For this purpose, GST-p12 proteins precipitated from mitotic (nocodazole-treated) and interphase (aphidicolin-treated) cell lysates were analysed by SDS-PAGE followed by sequential staining with PQ and SR dyes. From the PQ/SR ratios, GST-p12_WT and GST-p12_M63I were observed to be at least ~3–4 fold more phosphorylated at mitosis in comparison to interphase (Fig 6E, lanes 1 and 2 vs 3 and 4), which correlated well with the increased chromatin interaction observed for p12_M63I during mitosis. The S61A mutant was included as a negative control for phosphorylation.

### Phosphorylation increases affinity of recombinant Mo-MLV p12 M63I for chromatin

As both chromatin interaction and phosphorylation of GST-p12_M63I appear to increase in mitosis, we next investigated whether phosphorylation directly influences the chromatin interaction of p12 by exploring the effects of kinase inhibition and phospho-ablative/-mimetic mutations on histone precipitation.

**Fig 7:**
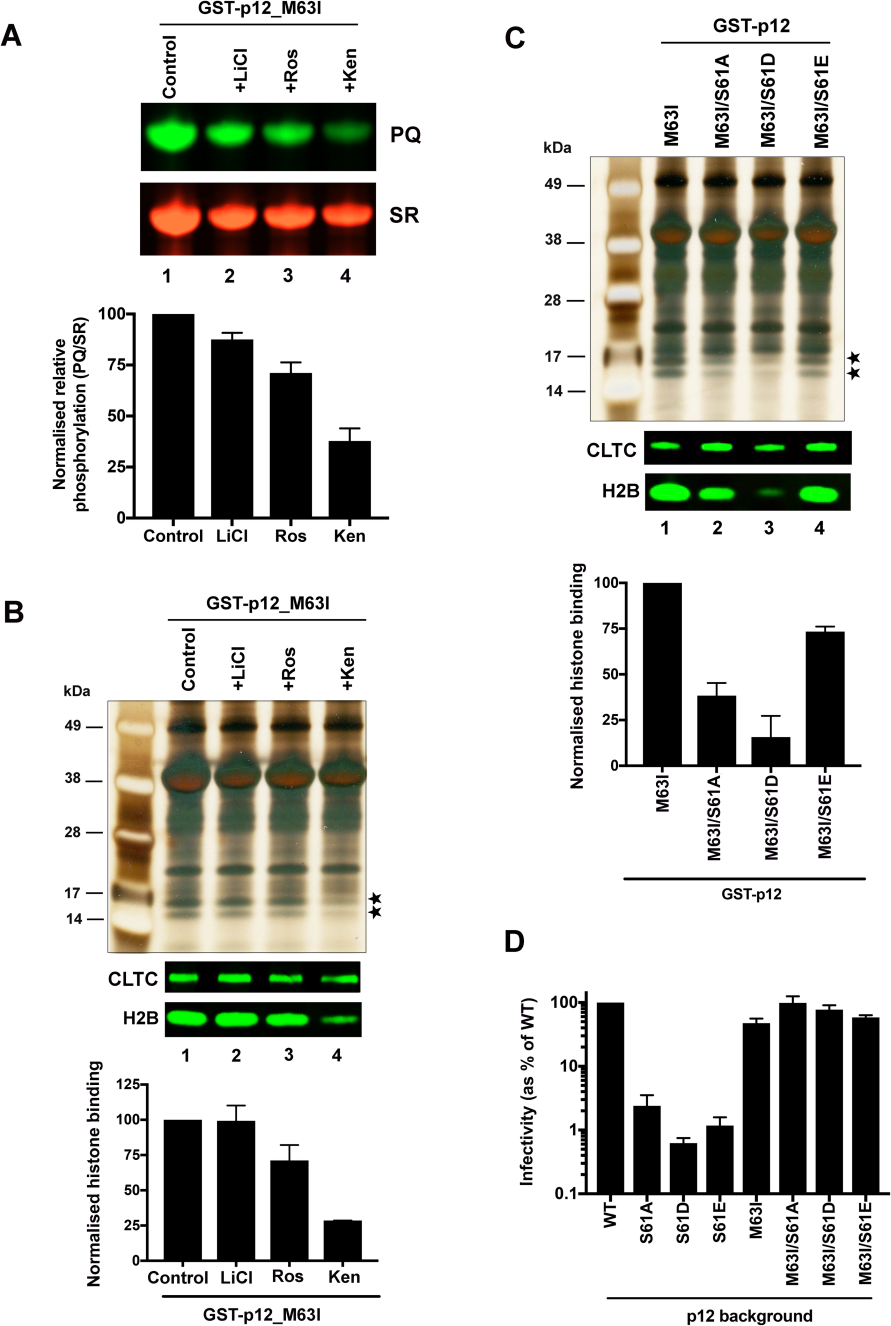
GST-tagged Mo-MLV p12_M63I has a higher affinity for chromatin when phosphorylated. (A and B) The effect of kinase inhibitors on p12 phosphorylation (A) and chromatin association (B). 293T cells transiently-expressing GST-p12_M63I were treated overnight with nocodazole, followed by a kinase inhibitor (LiCl, roscovitine (Ros) or kenpaullone (Ken)) for 3.5 h in the presence of both nocodazole and MG132, before lysis. Normalised cell lysates were incubated with glutathione-sepharose beads, bound proteins were separated by SDS-PAGE and gels were analysed either by sequential staining with ProQ diamond (PQ) and Sypro ruby (SR) dyes (A), or by silver-staining and immunoblotting with anti-CLTC and anti-H2B antibodies. PQ/SR ratios (A) and median H2B band intensities (B) are plotted in the bar charts as mean ± SD, of three technical replicates. (C) Mitotic chromatin association of GST-p12_M63I, S61 double mutants. 293T cells transiently-expressing GST-p12_M63I +/- an S61 mutation (S61A, S61D or S61E), were treated overnight with nocodazole and analysed as in (B). (D) Infectivity of Mo-MLV VLPs carrying alterations in p12. HeLa cells were challenged with equivalent RT units of *LacZ*-encoding VLPs carrying Mo-MLV p12_WT or M63I, +/- S61 mutations (S61A, S61D or S61E), and infectivity was measured 72 h post-infection by detection of beta-galactosidase activity in a chemiluminescent reporter assay. The data are plotted as percentage of WT VLP infectivity (mean ± SEM of >3 biological replicates).

Glycogen synthase kinase 3 (GSK3) and cyclin-dependent kinase 5 (CDK5) were predicted to phosphorylate Mo-MLV p12 on S61, by the NetPhos 3.1 kinase prediction algorithm [40, 41]. LiCl and roscovitine have been shown to inhibit GSK3 and CDK5 respectively, whereas kenpaullone inhibits both enzymes [42]. These drugs were tested in our biochemical assays for their ability to reduce GST-p12_M63I phosphorylation and chromatin association. In our assays, cells transiently-expressing GST-p12_M63I were cultured in media containing nocodazole overnight, prior to the addition of the kinase inhibitors. Kinases were only inhibited for a short period of time (3.5 h) in the presence of both nocodazole and MG132 (proteasome inhibitor), to avoid both cell lethality and exit from mitosis. After removal of the drugs, cells were immediately washed and lysed for glutathione-sepharose bead pull-down assays. The eluates from the beads were analysed by SDS-PAGE followed by sequential staining with PQ and SR dyes to quantify the phosphorylation levels of GST-p12_M63I. LiCl treatment had little effect on GST-p12_M63I phosphorylation (Fig 7A, lane 2). Roscovitine and kenpaullone reduced phosphorylation by ~1.5-fold and ~3-fold respectively (Fig 7A, lanes 3 and 4). To correlate GST-p12_M63I phosphorylation with its ability to interact with chromatin, we quantified the amounts of H2B in the pull-down eluates by immunoblotting. Whereas LiCl treatment had no significant effect on chromatin pull down (Fig 7B, lane 2), roscovitine and kenpaullone reduced H2B precipitation by ~1.5-fold and ~3.5-fold, respectively (Fig 7B, lanes 3 and 4), reflecting the observed decrease in phosphorylation. The short period of kinase inhibition used in our assays, may have limited the effects observed on GST-p12_M63I phosphorylation. However, our results suggest that reduction of p12 phosphorylation decreases its affinity for chromatin.

To explore the relationship between p12 phosphorylation and chromatin interaction further, we made phosphoablative (S61A) and ‘phosphomimetic’ (S61D and S61E) mutations in GST-p12_M63I. When tested in glutathione-sepharose bead precipitation assays, the S61A mutant showed an ~3-fold reduction in histone pull down (Fig 7C, lane 2). The decrease in chromatin association due to the loss of phosphorylation was partly rescued in the S61E mutant (~1.5-fold defect) but not in S61D (~6-fold defect) (Fig 7C, lanes 3 and 4). The extent to which aspartic acid and glutamic acid mimic the effect phosphorylation is heavily context-dependent, as these amino acids differ from phosphate in charge (-1 *versus* -1.5) and the number of oxygen atoms available for hydrogen bonding. In the context of the p12 protein, S61E might be better tolerated than S61D, as glutamic acid is more similar in size and geometry to phosphorylated serine than aspartic acid [43]. We also tested the effects of these phosphoablative and ‘phosphomimetic’ mutations on Mo-MLV infectivity. In the p12_M63I background, all three mutations increased infectivity slightly, by ~2-fold or less (Fig 7D). This was not surprising as the M63I mutation was initially described to be a compensatory mutation for loss of phosphorylation. However, in p12_WT, all S61 mutations decreased infectivity by >40-fold (Fig 7D), suggesting that S61 may make important contributions to the chromatin interaction beyond phosphorylation.

### The Mo-MLV p12 M63I interactome reveals similar chromatin binding partners to the PFV *h*CBS

**Fig 8:**
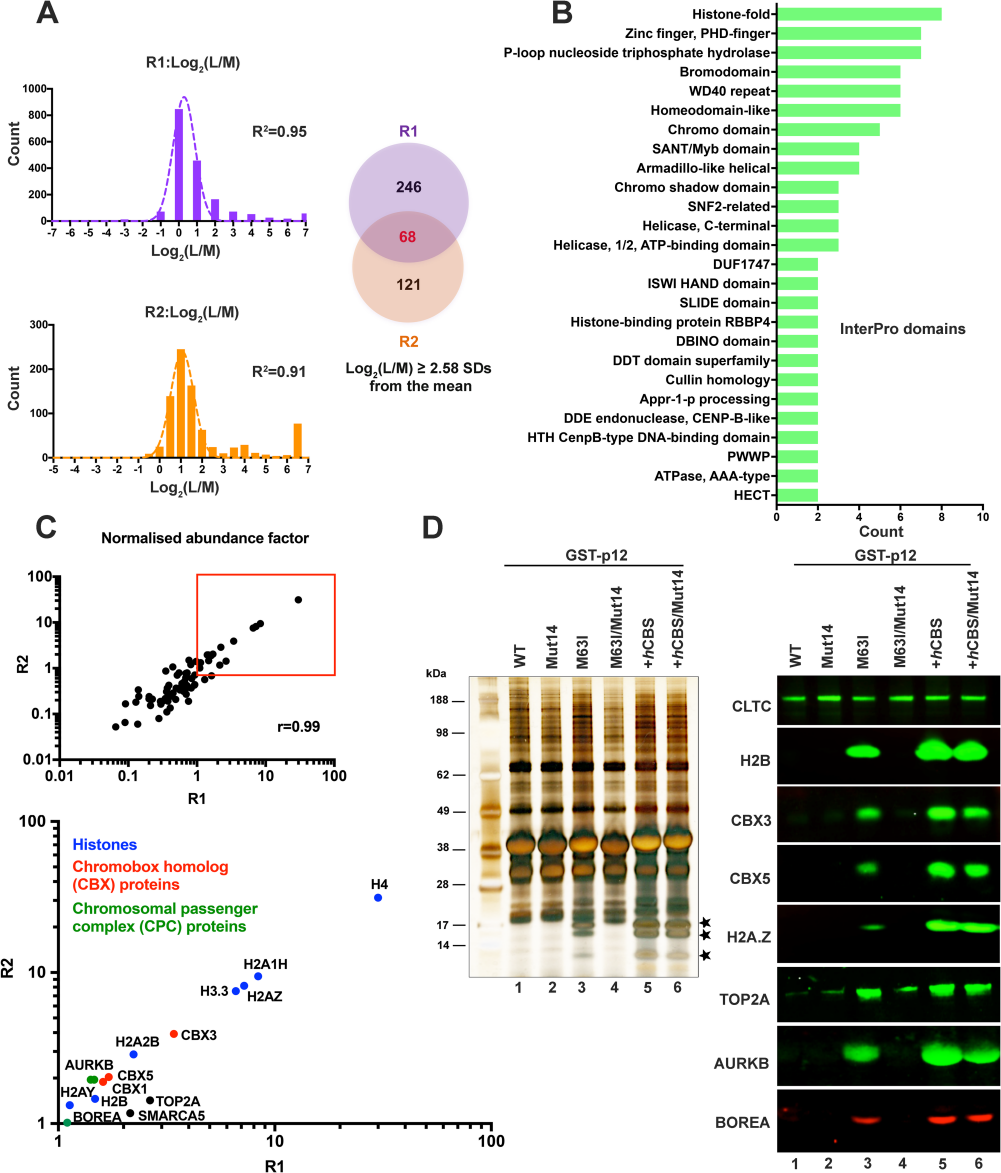
GST-p12_M63I interacts with the same chromatin-associated proteins as PFV CBS. Cellular proteins interacting with GST-p12_M63I were identified using SILAC-MS. Two biological repeats (R1 and R2) were performed. GST-p12_M63I and GST-p12_WT were transiently expressed in 293T cells cultured in light (R0/K0) or medium (R6/K4) SILAC media respectively. Cells were treated with nocodazole for mitotic enrichment and then lysed for glutathione-sepharose bead pull-down assays followed by MS. (A) Identification of proteins enriched in the light-labelled GST-p12_M63I (L) sample relative to medium-labelled GST-p12_WT (M) sample. Log_2_(L/M) silac ratios of the set of MS hits (FDR <5%) from each replicate were plotted as a frequency distribution. Mean and standard deviation (SD) of each distribution was estimated by fitting to a normal distribution curve (R^2^≥0.91). MS hits with log_2_(L/M) ratios greater than 2.58 SDs from the mean were selected as significantly enriched in the GST-p12_M63I (L) sample. Of the 314 such proteins identified in R1, 68 were also found in R2 (Venn diagram). (B) Functional classification of the 68 GST-p12_M63I interacting proteins in (A) was performed based on InterPro domain annotation. (C) The abundance factor for each protein was calculated by dividing the peptide spectral count by its length. These values were then normalised across the group and the scores for R1 plotted against the scores for R2 (Pearson correlation *r* = 0.99). MS hits with normalised abundance factors ≥1 in both replicates are boxed in red (top panel) and expanded in the bottom panel. The points are coloured according to the protein function shown in the key: Blue, nucleosomal histone proteins; Red, chromobox homolog proteins; Green, chromosomal passenger complex proteins; Black, others. (D) Validation of SILAC results. 293T cells were transiently-transfected with expression constructs for GST-tagged Mo-MLV p12_WT (lane 1), p12_mut14 (lane 2), p12_M63I (lane 3), p12_M63I/mut14 (lane 4), p12+*h*CBS (lane 5) and p12+*h*CBS/mut14 (lane 6) and GST pull-down assays were performed and analysed as in Fig 6. Immunoblots (right hand panels) were probed with a selection of antibodies to host proteins of interest (S4 Table). Bands corresponding to core histones in the silver-stained gel are starred.

GST-p12_M63I was seen to colocalise with mitotic chromatin and to precipitate histones in GST-pull down assays (Figs 5 and 6). However, it could be interacting directly with components of chromatin or indirectly via other chromatin-binding proteins. To potentially identify the target of p12_M63I, we compared the interactome of GST-p12_M63I (which showed chromatin association) to that of GST-p12_WT (which did not) in 293T cells, using a SILAC-MS approach (Fig 8).

Light (R0/K0) and medium (R6/K4) SILAC-labelled cells transiently expressing GST-p12_M63I and GST-p12_WT respectively were treated with nocodazole for mitotic enrichment and then lysed for glutathione-sepharose bead pull-down assays. Eluates were subsequently pooled in a 1:1 ratio for analysis by LC-MS/MS. As before, the experiment was performed using two biological replicates to test reproducibility. The mass-spec hits identified at 5% FDR were subjected to further downstream analysis, in order to select proteins significantly enriched in the light-labelled (L) GST-p12_M63I sample relative to the medium-labelled (M) GST-p12_WT sample. For 68 of these proteins the log_2_(L/M) ratios were greater than 2.58 standard deviations (SDs) from the mean (99% confidence threshold) in both replicates, suggesting that they may interact with GST-p12_M63I (Fig 8A, S2 Table). Interestingly, InterPro domain analysis [44] of these proteins revealed the vast majority to be involved in the regulation of chromatin structure and function (Fig 8B). Furthermore, when ranked on normalised abundance within the group, the highest-scoring hits in both replicates were nucleosomal core histones. The other top-ranking hits, with a normalised abundance ≥1 in both replicates, included chromobox homolog proteins and components of the chromosomal passenger complex (Fig 8C). To validate the mass-spectrometry results, we next performed pull-down assays with mitotic cell lysates containing GST-tagged p12_WT, p12_mut14, p12_M63I, p12_M63I/mut14, p12+*h*CBS and p12+*h*CBS/mut14 proteins. The eluates from the assays were analysed by silver-staining and immunoblotting for the top protein hits for GST-p12_M63I in the mas-spec analysis (Fig 8D). All proteins tested were precipitated by p12+*h*CBS and p12+*h*CBS/mut14 as well as p12_M63I (Fig 8D, lanes 3, 5 and 6). However, none were precipitated with the negative controls p12_mut14 and p12_M63I/mut14 (Fig 8D, lanes 2 and 4), or the p12_WT, protein (lane 1).

As p12_M63I and PFV *h*CBS showed similar chromatin interactions (Fig 8D), we performed another SILAC-MS experiment to compare their global interactomes. GST-p12+*h*CBS/mut14 was used to ensure that all of the observed interactions were specific to the *h*CBS and that there was no contribution from the CTD of p12. Both GST-p12_M63I and GST-p12+*h*CBS/mut14 pull-down eluates were compared to GST-p12_M63I/mut14 pull-down eluates, to identify their putative chromatin interactions. Of the 73 proteins identified as GST-p12_M63I interactors from this analysis, ~90% (including 39 of the 40 top-ranked hits) overlapped with the GST-p12+*h*CBS/mut14 interactome (S4 Fig). Together with the observation that the top SILAC-MS hits for p12_M63I were core histones, this suggests that p12_M63I may also bind chromatin directly like the PFV *h*CBS [14, 15].

### Gammaretroviral p12 directly binds recombinant nucleosomes *in vitro*

**Fig 9:**
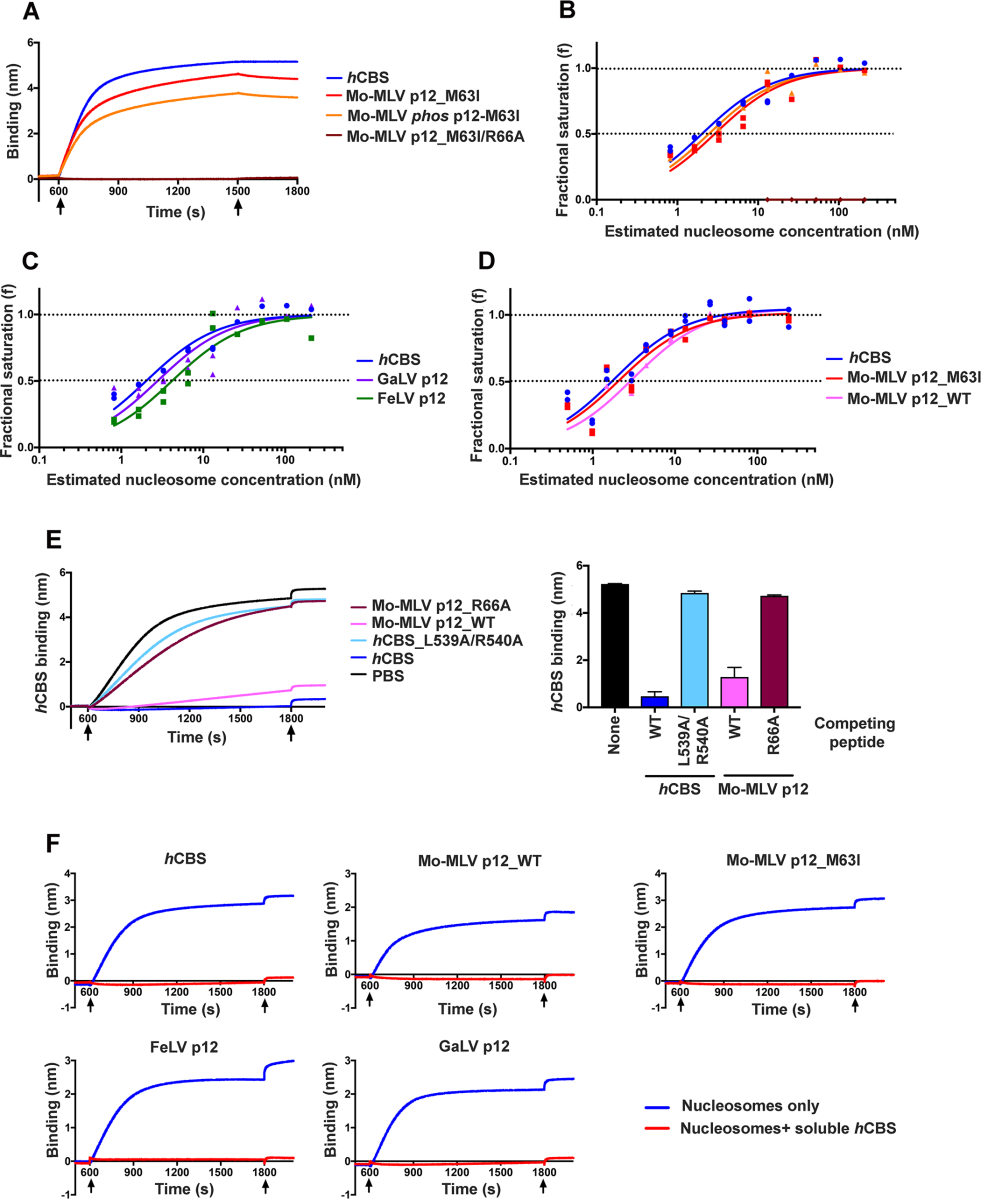
The p12 CTD directly binds recombinant nucleosomal arrays. Direct interactions between p12 CTD peptides and nucleosomal histone proteins was tested using biolayer interferometry (BIL). Streptavidin sensor probes were coated with biotinylated p12 CTD or *h*CBS (positive control) peptides to equivalent levels and then immersed in analyte solutions containing recombinant poly-nucleosomes to measure binding. (A) Representative sensorgrams showing binding of peptides corresponding to the CTD of Mo-MLV p12_M63I, *phos* p12-M63I (S61-phosphorylated), p12_M63I/R66A or *h*CBS to recombinant poly-nucleosomes (~250 nM). Black arrows indicate the beginning and end of the association phase. (B-D) Affinity measurements were derived by probing peptides corresponding to *h*CBS (B-D) or the CTD of Mo-MLV p12_M63I, *phos* p12-M63I (B), GaLV p12, FeLV p12 (C) or Mo-MLV p12_WT (D) against a range of serially-diluted nucleosomes. Equilibrium binding data from two technical replicates were pooled for the analysis. The plotted data are fractional saturation of binding as a function of nucleosome concentration. (E) Competition assay inhibiting nucleosome binding to immobilised biotin-tagged *h*CBS with soluble non-biotinylated *h*CBS or Mo-MLV p12 CTD peptides as indicated in key (100 μM). Left panel shows representative sensorgrams with black arrows indicating the beginning and end of the association phase. The bar chart shows equilibrium binding of *h*CBS in the presence and absence of competing peptides as mean ± SD from three technical replicates. (F) Representative sensorgrams showing competition assay inhibiting nucleosome binding to immobilised biotin-tagged p12 CTD peptides or *h*CBS, as indicated in graph title, with soluble non-biotinylated *h*CBS (100 μM, red lines). Black arrows indicate the beginning and end of the association phase. See methods for peptide sequences.

The PFV *h*CBS is known to bind to the nucleosomal core histones H2A and H2B [14, 15]. To identify whether p12 proteins also interact directly with nucleosomes, we used biolayer interferometry (BLI) to probe the binding of recombinant poly-nucleosomal arrays by p12 CTD peptides (Fig 9). Streptavidin sensor probes coated with biotinylated peptide ligands were immersed in ‘analyte’ solutions containing the *in vitro* assembled nucleosomal arrays to observe any potential interactions. The p12 CTD peptide sequences used are highlighted in bold in Fig 5C. A peptide carrying the PFV *h*CBS sequence was included in the assays as a positive control.

We first tested the binding of Mo-MLV p12_M63I CTD peptides to nucleosomes (Fig 9A). Excitingly, both S61-phosphorylated and non-phosphorylated p12_M63I peptides showed clear binding to nucleosomes (Fig 9A, orange and red lines). This binding was specific as an interaction was not observed with the negative control p12_M63I_R66A peptide (Fig 9A, dark red line). R66 is part of the ‘SRLRG’ motif in the p12 CTD which is required for tethering to mitotic chromatin (Fig 2B and Fig 8D) and we have previously shown that Mo-MLV VLPs carrying p12_R66A are non-infectious [7]. To compare the affinities of PFV *h*CBS and phosphorylated/non-phosphorylated p12_M63I for nucleosomes, the peptides were also tested against a dilution series of the nucleosomal preparation (Fig 9B). The equilibrium dissociation constant (K_d_) measurements derived from this experiment were in the low nanomolar range (~2–3 nM) for all peptides, indicating that the peptides bound very strongly to the nucleosomes. Furthermore, the estimated K_d_ measurements for *h*CBS, phosphorylated p12_M63I and non-phosphorylated p12-M63I peptides in this experiment were very similar (<2-fold variance). Although somewhat surprising, the low Kd values may arise from the high avidity of the polymeric nucleosome arrays (up to 11 nucleosomes per DNA molecule) used.

Compellingly, p12 CTD peptides from FeLV and GaLV, two gammaretroviruses that also demonstrated chromatin interactions in our GST pull-down assay (Fig 5D), also showed high affinity (nM) binding to recombinant nucleosomal arrays (Fig 9C). Although we could not detect any interactions of GST-p12_WT with chromatin in our previous assays, we reasoned that the high sensitivity of BLI coupled with the high avidity of our polymeric nucleosome arrays may sufficiently compensate for a potential low affinity interaction between Mo-MLV p12_WT and chromatin allowing us to detect binding. Indeed, provokingly, a biotinylated peptide corresponding to the CTD sequence of Mo-MLV p12_WT showed measurable binding to nucleosomal arrays by BLI (Fig 9D). This suggests that WT p12 does bind directly to chromatin *in vitro* but that the affinity is too low to detect the interaction in most standard assays.

To identify whether p12 binds to the same nucleosomal docking site as the CBS from PFV Gag, we then tested the ability of p12 CTD peptides to block the binding of nucleosomal arrays to *h*CBS and *vice versa* (Fig 9E and 9F). In the first set of these experiments, nucleosomal arrays were pre-incubated with excess non-biotinylated Mo-MLV p12_WT, Mo-MLV p12_R66A, *h*CBS or *h*CBS_L539A/R540A peptides, prior to recording BLI measurements with biotinylated *h*CBS immobilised on sensor probes (Fig 9E). L539A/R540A mutations have previously been observed to prevent binding of *h*CBS peptides to nucleosomes [15]. Equilibrium binding of nucleosomes to immobilised *h*CBS was significantly decreased in the presence of both soluble *h*CBS (by ~10-fold) and p12_WT (~4-fold), but not *h*CBS_L539A/R540A or p12_R66A peptides (Fig 9E). In reciprocal experiments, a soluble non-biotinylated *h*CBS peptide could compete with the binding of immobilised Mo-MLV p12_WT, Mo-MLV p12_M63I, FeLV and GaLV to the nucleosomal arrays (Fig 9F). Overall, these results suggest a conserved mode of chromatin binding between spuma and gamma retroviruses.

## Discussion

It has long been known that the Gag-cleavage product p12 is essential for MLV replication [6]. As well as the late-domain required for budding, p12 contains N- and C-terminal functional domains that are required for the early stages of replication [6, 7]. We previously showed that p12 binds CA and that mutating the NTD of p12 results in reduced stability and abnormal morphology of viral cores leading to reduced infectivity [9]. Additionally, mutating the CTD of p12 prevents viral PICs from associating with chromatin and reduces infectivity to less than 1%. The infectivity of p12 CTD mutants can be partially restored by inserting a heterologous CBS into p12 [7, 11, 12]. Here, we confirm and extend these observations and provide further mechanistic insights into the functions of both N- and particularly C-terminal p12 domains.

In this study, we showed that the NTD of p12 interacts specifically with a regular hexameric arrangement of CA and not individual CA monomers *in vitro* (Fig 1). Moreover, we were also able to detect an association of p12 and CA in mature viral particles by co-immunoprecipitation. Mutations to the CTD of p12 did not disrupt this interaction, either in viruses or in *in vitro* binding assays, suggesting that the CTD of p12 does not make CA interactions, and that any potential NTD-CTD interactions within p12 are not required for CA binding. Importantly, this also implies that CA binding does not modify the CTD of p12 directly. Specific binding to mature CA lattices supports previous findings that immature particle assembly is not affected by p12 mutations [9], and that in a NMR study of a Gag-like fragment, no long-range interactions could be detected between p12 and the N-terminal region of CA [33]. Intriguingly, we calculated a p12:CA binding ratio of 1:6 from our *in vitro* binding experiments (S1 Fig) that may be indicative of p12 binding to hexameric rings of CA monomers. However, this may be an under-estimate, as p12 may not occupy all available sites on the lattice, even when present in molar excess, and it is possible that not all the CA on the lipid tubes will form regular hexamers [26]. Further studies will be required to identify the p12 binding site on CA, which, in turn, may reveal how p12 stabilises the CA shell.

Interestingly, we observed co-localisation of CA with p12 in infected cells (Fig 2), even on mitotic chromatin. Notably, our findings differ from a previous study in which co-localisation of CA with p12 was observed in the cytosol of infected cells but not on mitotic chromatin [11]. However, as the antibodies and fixative procedures used in that study are different from the ones used here, we believe that the discrepancy in the observations could be due to different sensitivities of CA detection. The CA shell of HIV-1 is proposed to disassemble before integration. Although the timing of such “uncoating” is controversial, it appears to be triggered after the first strand transfer step of reverse transcription [45]. Little is known about the uncoating of other retroviruses, but MLV CA has also been observed to dissociate from the PIC gradually with time [9]. This implies that the amount of CA still associated with the PIC upon chromatin tethering is likely to be less than in the viral core, making it harder to detect. We previously found that the fixative procedures for the anti-CA and anti-p12 antibodies were not compatible, and we therefore stained for p12 and CA on separate slides [9]. Here, we introduced a two-step fixation procedure (4% PFA and methanol) and optimised membrane permeablisation of our samples, which now allows us to robustly detect both p12 and CA simultaneously. When we introduced mutations into the CTD of p12, the protein remained co-localised with CA but no longer associated with chromatin. In contrast, mutating IN to prevent interaction with BET proteins had no effect on CA or p12 localisation. This implies that CA association with mitotic chromatin is primarily driven by p12.

MLV CA is a component of the PIC [46] but whether it plays a role in the chromatin targeting of the viral genome like HIV CA [47] is currently not known. Recently the interaction of MLV IN with BET proteins was shown to mediate integration site selection in infected cells [20–22]. We showed here that VLPs carrying an IN mutant deficient in BET binding were only mildly defective (<2-fold) in infectivity compared to WT virus, whereas p12 CTD mutant VLPs were less than 1% infectious. These results suggest that p12 targeting to mitotic chromatin is mandatory for integration, and likely occurs prior to IN-chromatin binding. Enticingly, this is reminiscent of the proposed interplay between the HIV-1 CA-binding host protein, cleavage and polyadenylation specificity factor 6 (CPSF6) and IN-binding lens epithelium-derived growth factor (LEDGF/p75) interactions in the nuclear targeting of HIV-1 [47]. CPSF6 is currently thought to direct the HIV-1 PIC to transcriptionally-active chromatin, and then LEDGF/p75 mediates the local targeting of integration specifically into genes. MLV p12 could therefore play a similar role to CPSF6 in facilitating nuclear/chromatin delivery of CA-containing viral PICs. This analogy could even be partly extended to foamy viruses that have a CBS within their Gag shell. This would suggest an evolutionary conserved overall pathway for retroviral chromatin targeting, albeit with different mechanistic details for individual viruses. It will be interesting to investigate the timing of p12 and CA dissociation from each other, and from chromatin, to determine the order of events relative to integration itself.

Excitingly, we have shown for the first time that WT Mo-MLV p12 CTD, but not the defective R66A CTD mutant, binds directly to nucleosomal arrays *in vitro* (Fig 9). This shows that p12 tethers the PIC to chromatin via a direct interaction and not through another chromatin binding protein. Furthermore, it confirms that only the CTD motif is necessary for the binding. Nucleosome binding was also observed with p12 orthologues of FeLV and GaLV, suggesting that direct chromatin tethering is a common feature of gammaretroviruses. Importantly, p12 CTD peptides were able to compete with the binding of the PFV CBS to nucleosomal arrays, and *vice versa*, in BLI assays, indicating that they all bind around the same site. The similarity between gammaretroviral p12 and PFV CBS chromatin binding is supported by our data from cell-based assays. Global mass-spectrometric analysis of the chromatin interactome of p12_M63I revealed a very significant overlap of ~90% with that of the PFV CBS (Fig 8 and S4 Fig). The docking site of the PFV CBS on nucleosomal surfaces has been mapped by x-ray crystallography to an acidic patch at the H2A-H2B heterodimeric interface [15]. Interestingly, this is also the target site of the KSHV LANA protein on nucleosomes [18]. Both PFV CBS and KSHV LANA carry an arginine side chain that projects deep into the nucleosomal acidic pocket and makes critical contacts with its carboxylate groups. Gammaretroviral p12 proteins also carry a number of conserved arginine residues in the CTD which have been shown to be essential for infection and chromatin association (Figs 2, 8 and 9) [7], suggesting that p12 proteins bind the same nucleosome docking site using a conserved mechanism.

Our *in vitro* nucleosome binding assays recapitulated the mitotic chromosome tethering of Mo-MLV p12 seen in infected cells (Fig 2) [11]. However, in order to characterise this interaction better in cells, we initially studied GST-tagged p12 proteins. Although we showed that GST-tagged FeLV, GaLV and the Mo-MLV p12_M63I mutant could precipitate histones from cell lysates (Fig 5), surprisingly, we could not detect a chromatin association in cells with GST-tagged WT Mo-MLV p12 (Fig 3) or indeed other MLVs (N-tropic MLV and XMRV, Fig 5). There are a few possible explanations for this:

A previous study attributed the inability of a WT Mo-MLV p12-GFP fusion protein to bind chromatin to an absence of phosphorylation [19]. However, ~50% of the GST-tagged Mo-MLV p12_WT protein expressed in our cells was clearly phosphorylated on S61 (Fig 3, Table 1). The sensitivity of our assays would have allowed us to detect an interaction between p12 and chromatin in this fraction of p12, indicating that the absence of detectable chromatin binding here is not due to a lack of phosphorylation. However, phosphorylation may be involved in modulating the affinity of p12 for chromatin, as reducing phosphorylation of GST-p12_M63I using kinase inhibitors or an S61A mutation correlated with reduced chromatin binding (Fig 7). Moreover, p12_M63I had a higher affinity for chromatin in mitosis compared to interphase (Fig 6) [11], and this correlated with an increase in phosphorylation during mitosis. Surprisingly, the phospho-mimetic mutations S61D and S61E also reduced chromatin binding. However, changing these residues may affect the hydrogen bonding capacity or conformation of p12 as well as phosphorylation. Notably, a link between serine/threonine phosphorylation and chromatin binding has been established for the LANA protein of KSHV. Preventing LANA phosphorylation by short- term treatment (4 h) with a RSK inhibitor decreased H2B binding by ~50% [48]. Kinases which phosphorylate LANA also appear to phosphorylate the EBNA-1 protein of EBV, therefore chromatin binding of the latter may also be subjected to similar post-translational regulation [48]. Whether phosphorylation of LANA and EBNA-1 proteins is cell-cycle dependent is not known.

As the inability of Mo-MLV GST-p12_WT to bind chromatin was not due to the lack of its phosphorylation, we wondered whether the absence of other viral proteins, particularly CA, may be steering GST-p12 to behave like the p12 region of Gag. In Gag, p12 recruits the ESCRT proteins required for viral budding through the L-domain PPPY motif and interacts with the clathrin heavy chain (CLTC) via the DLL motif in the NTD. As well as promoting budding, binding of these cellular proteins may disfavour chromatin binding. When a virus enters a new target cell, it must travel towards the nucleus, and at this time it would not be advantageous to recruit these membrane-associated host factors. Binding of the p12 NTD to CA may prevent interactions with these host proteins and instead facilitate the early steps of infection.

Investigating the cellular interactome of Mo-MLV GST-p12_WT revealed that it did indeed bind WWP2 and CLTC (Fig 4). However, engineering p12 to remove the PPPY or DLL motifs, or the whole NTD, did not rescue chromatin tethering of GST-p12 (Fig 5 and S3 Fig). Thus, chromatin binding is not significantly facilitated by preventing other host proteins from interacting with p12. However, CA could increase the affinity of p12 for chromatin in alternative ways, perhaps by altering the confirmation of p12, or by providing supplementary interactions to increase the affinity of binding. In our assays, chromatin binding of Mo-MLV GST-p12_WT was rescued by the M63I substitution in the p12 CTD. As GST-p12 is phosphorylated, M63I must be doing more than just compensating for a lack of this modification in our system. Importantly, the chromatin binding of this mutant was still dependent on the residues altered in Mut14 (Fig 5), suggesting that the M63I change is enhancing the ability of p12 to interact with chromatin without altering its mode of binding. M63I, therefore, probably increases the affinity of p12 for chromatin via a conformational change. This change may, at least in part, mimic the effect of CA-binding in the context of the viral PIC. The ability of M63I to rescue both phosphorylation-deficiency [25] and an absence of CA binding in the presence of phosphorylation (Fig 6), suggests a possible functional overlap between these two processes in enhancing chromatin binding of p12.

Another way in which CA could facilitate the chromatin tethering of the p12 CTD is by simply elevating the local concentration of p12 to increase the avidity of its interactions. The presence of multiple nucleosomes on the polymeric arrays used in our *in vitro* assay (up to 11 per DNA) may also increase the avidity of binding, allowing us to observe an interaction between WT Mo-MLV p12 that we did not observe in cells. A high avidity of polysomes could also explain why the different gammaretroviral p12 peptides had similar Kd values to each other and the CBS peptide in our BLI assays (Fig 9), despite the CBS showing greater histone binding in pull-down assays (Fig 5).

**Fig 10:**
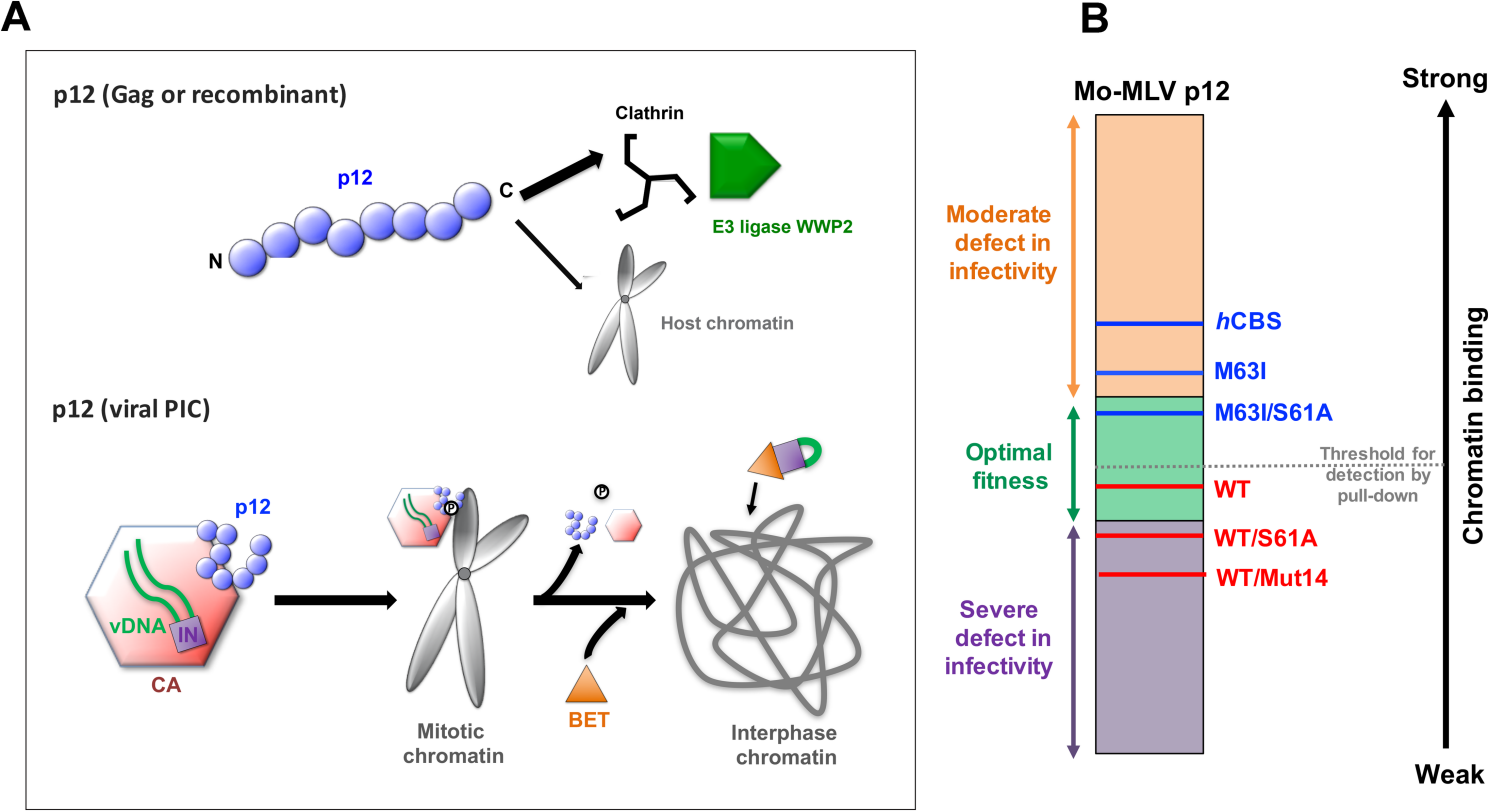
Models for p12-chromatin binding. (A) Proposed model for the different functions of p12. The p12 region of Gag and p12 protein in the viral PIC differ in their affinity for cellular proteins and chromatin. We propose that as part of Gag, or when expressed as a recombinant GST-fusion protein, p12 exists in an unstructured conformation with low affinity for nucleosomes but relatively high affinity for host proteins such as clathrin and NEDD4-like E3 ligases which facilitate late replication events. Following Gag cleavage, the binding of the p12 NTD to the CA lattice promotes a change in the conformation of p12 which increases the affinity of the p12 CTD for nucleosomes. During mitosis, the breakdown of the nuclear envelope allows the p12/CA-containing PIC to access chromatin. The PIC is targeted to nucleosomes on mitotic chromatin by CA-bound, phosphorylated p12. Exit from mitosis promotes the de-phosphorylation of p12 and the dissociation of p12 and CA from chromatin. BET proteins can then bind IN and direct the viral cDNA to gene promoter regions where integration occurs. (B) Proposed relationship between virus infectivity and affinity of p12 for chromatin. We suggest that the affinity of p12 for chromatin is fine-tuned for optimal infectivity with deviations incurring a fitness cost. Mutations in p12 that increase or decrease chromatin binding (measured, in blue, or extrapolated, in red) alter viral infectivity as shown on the left. Only interactions above an arbitrary threshold can be detected by GST-pull down assays.

Based on the results from this study and previous observations, we propose a model for the chromatin binding function of gammaretroviral p12 as shown in Fig 10A. As part of Gag, p12 exists in a largely unstructured conformation with high affinity for host proteins which facilitate viral budding but relatively low affinity for nucleosomes. Following Gag cleavage, p12 binds to the hexameric CA lattice via its NTD, stabilising the viral core and causing a change in the conformation of p12 which increases the affinity of p12 for nucleosomes. Upon breakdown of the nuclear envelope in mitosis, the PIC is targeted to condensed chromatin by CA-bound, phosphorylated p12. Following exit from mitosis, either dephosphorylation of p12 or chromatin decondensation could reduce the affinity or avidity of the interaction and cause p12 to be released from chromatin. This may also release CA from the PIC and expose the viral cDNA and IN to chromatin. BET proteins then bind IN and direct the intasome to promotor regions where integration occurs. Failure of p12 to interact with chromatin leads to a severe replication defect (<1% infectivity, Fig 2). This may be partly due to exclusion from the re-assembled nucleus of unbound PICs, but likely indicates that p12-chromatin binding is required for an essential step preceding integration, like CA uncoating. Interestingly, in agreement with previous findings by Schneider *et al*. [12], we have observed that alterations to the Mo-MLV p12 protein that increase the affinity for chromatin to a level that enables the interaction to be detected in microscopy or pull-down assays have a modestly detrimental effect on infectivity (Fig 5). These include the M63I substitution and the insertion of the PFV *h*CBS. Therefore, increased binding of p12 to chromatin may result in integration at the initial site of chromatin contact and prevent BET proteins targeting IN to optimal sites for integration. These results suggest that chromatin binding of gammaretroviral p12 is fine-tuned for optimal affinity and that stronger or weaker binding both appear to incur a fitness cost (Fig 10B).

In conclusion, we have demonstrated that gammaretroviral p12 docks to mitotic chromatin via a direct interaction with nucleosomal histones, similarly to spumaviruses. The chromatin binding of p12 is likely facilitated by binding to CA after viral maturation and by phosphorylation of p12 in mitosis. By tethering the CA-containing PIC to mitotic chromatin, p12 may influence global nuclear targeting of MLV integration, similarly to the proposed role of the CA-binding host protein CPSF6 in HIV-1 infection. Many retroviral genera have additional Gag-cleavage products in a similar genomic position to p12, mostly with unknown functions but often containing a Late-domain. It is possible that they too have chromatin binding sequences and guide their viral PICs to chromatin. The efficiency of integration and of proviral transcription varies amongst retroviruses and it is tempting to speculate that this is due to differences in the affinity of such chromatin binding sequences for chromatin. Further work is needed to assess whether p12 function is a conserved general feature of retroviral replication.

## Materials and methods

### Plasmids and cloning

For bacterial expression, N-MLV p12 sequences (WT, Mut6 and Mut14) were amplified from pCIG3N [7] and cloned into pGEX6.1 using BamHI/XhoI sites. C-terminally His-tagged N-MLV CA WT and P1G mutant proteins were expressed from pET22-N-MLV-CA plasmids as previously described [26].

Retroviral VLPs were synthesised by co-transfection of three plasmids: an envelope expression plasmid for vesicular stomatitis virus G protein (pczVSV-G) [49], a Mo-MLV-based retroviral vector encoding *LacZ* (pczLTR-LacZ) [50] and a Gag-Pol expression plasmid for either Mo-MLV (pKB4) [7] for infectivity and microscopy assays or Mo-MLV with myc-tagged p12 (pKB4mycE) [9] for viral Co-IP assays. The generation of p12 alanine-scanning mutations in pKB4 and pKB4mycE has been described previously [7, 9]. The M63I, G49R/E50K, S61A/D/E changes were introduced into Mo-MLV p12 and the W390A mutation was introduced into IN by mutagenesis of pKB4, using the QuikChange II site-directed mutagenesis kit (Agilent technologies) and primers shown in S3 Table.

GST-p12 fusion proteins were expressed from pCAGGS/GST-derived plasmids. pCAGGS/GST carrying WT Mo-MLV p12 was a kind gift from J. Martin-Serrano [5]. Mo-MLV p12 mutants (Mutants 6 to 15, L-dom, p12+*h*CBS) were sub-cloned into pCAGGS/GST from the Mo-MLV Gag-Pol vector, pKB4 [7] using EcoRI restriction sites. N-MLV p12 was similarly sub-cloned from pCIG3N [7]. Codon-optimised FeLV, GaLV, XMRV and KoRV p12 sequences were synthesised by GeneART (Thermo Fisher Scientific) and then cloned in to pCAGGS/GST using EcoRI/XhoI sites. The Mo-MLV p12 mutants M63I, G49R/E50K, D25A, S61A/D/E as well as the FeLV p12 mutants I52M and A53V were generated by site-directed mutagenesis of the pCAGGS/GST plasmids using primers shown in S3 Table. The Mo-MLV p12 CTD was amplified from pKB4 using primers: for 5’-atgaattcggagaggcaccggacc-3’ and rev 5’-tggaattcaagggggctcccgtctc-3’, and cloned into pCAGGS/GST using EcoRI sites. To generate stable GST-p12 expressing lines, GST-p12 sequences were amplified from pCAGGS/GST plasmids and sub-cloned into a Mo-MLV-based retroviral vector encoding puromycin-resistance called pCMS28 [51] using BglII/XhoI restriction sites. Transduction vectors were synthesized by cotransfection of pczVSV-G, pKB4 and pCMS28/GST-p12.

HA-tagged IN was expressed from pCMV4/HA which was generated by cloning a codon-optimised IN sequence (synthesised by GeneART, Thermo Fisher Scientific) into the pCMV4-HA vector [52] using MluI/SbFI restriction sites.

### Cells and VLP production

HEK 293T, HeLa and D17 cells (Bishop laboratory cell stocks) were maintained in DMEM (Thermo Fisher Scientific), supplemented with 10% heat-inactivated foetal calf serum (Biosera) and 1% penicillin-streptomycin (Sigma). The cells were stored in a humidified incubator at 37°C and 5% CO_2_.

VLPs were made by co-transfecting 293T cells with plasmids encoding VSV-G (pczVSV-G), Mo-MLV Gag-Pol and the LacZ reporter gene (pczLTR-LacZ), in an equimolar ratio. Approximately 16 h after transfection the cells were treated with 10 mM sodium butyrate for 6 h to promote transcription. VLP-containing culture supernatants were harvested 48 h post-transfection and filtered to remove cellular debris. Viral titres were quantified using a modified ELISA for reverse transcriptase activity (Cavidi). Transduction vectors for stable cell line generation were produced by co-transfecting 293T cells with plasmids encoding VSV-G (pczVSV-G), Mo-MLV Gag-Pol (KB4) and GST-p12, as described above. HeLa cells were transduced with these VLPs by spinoculation (1600 g, 2 h, 16°C) in the presence of 4 μg/ml polybrene (Sigma). From approximately 72 h after infection, cells were passaged in media containing 0.5 μg/ml puromycin (Thermo Fisher Scientific) to select for transduction.

### Bacterial protein expression and purification

The N-terminally GST-tagged p12 fusion proteins were produced in *E*. *coli* Rosetta 2(DE3)pLysS cells (Thermo Fisher Scientific) from pGEX.1-derived plasmids. The cells were grown in the presence of 1% glucose and GST-p12 expression was induced by the addition of 1 mM isopropyl-β-D-thiogalactopyranoside (IPTG) in mid-log phase. Cells were subsequently lysed in 50 mM Tris pH 8, 500 mM NaCl, 0.5 mM TCEP, 0.1% Triton X-100 (Buffer A) in the presence of protease inhibitors (Roche) and incubated with Lysozyme (Sigma Aldrich) and Benzonase (Sigma Aldrich) for 1 hour at 4°C. Lysis was facilitates by sonication, x2 pulses, 5 minutes, 40% amplitude and crude lysates were centrifuged at 48,000 g, 45 minutes, 4°C to remove debris. The clarified lysates were applied to 1 ml GST-trap columns (GE Healthcare). After washing with Buffer A, untagged-p12 was eluted from the resin by digestion with 3C precision protease (GE Healthcare). The eluate was then heated at 65°C for 10 minutes and centrifuged at 40,000 g for 20 minutes to remove precipitates. Acetic acid (pH ∼3) was added to the supernatant which was then centrifuged at 40,000×g for 20 minutes to remove nucleotides and DNA. The supernatant was then applied to a Superdex 75 (16/60) size exclusion column equilibrated in 200 mM Ammonium bicarbonate. Eluate fractions containing p12 were pooled and lyophilised. The purity of the protein preparations was assessed by SDS-PAGE and the concentrations were determined from the absorbance at 280 nm. C-terminally His-tagged N-MLV CA WT and P1G mutant proteins were expressed and purified as previously described [26].

### Binding assays to purified CA

Binding of purified p12 to *in vitro* assembled CA arrays was performed essentially as previously described [26]. Briefly, lipid nanotubes were synthesised using the tube-forming lipid, d-galactosyl-β-1,1′ N-nervonoyl-d-erythro-sphingosine (GalCer) (Avanti) in combination with the Ni^2+^-chelating lipid, DGS-NTA (Avanti) in a 7∶3 ratio. After mixing the lipids, residual chloroform and methanol were removed under a gentle stream of nitrogen and the lipids were resuspended with the aid of sonication in 10 mM Tris-HCl pH 8, 10 mM KCl, 100 mM NaCl, to a final concentration of 0.5 mg/ml. The tubes were coated by incubating with 2 mg/ml of purified His-tagged N-MLV wild type CA or P1G CA mutant at a ratio of 1∶3 with 10 mM imidazole, for 1 hour at room temperature. To assess binding of p12 to non-hexameric CA, purified 2 mg/ml CA was also immobilised on HIS-Select Nickel Affinity beads (Sigma) at a 1:10 ratio. Purified p12 proteins were diluted to approximately 50 μg/ml, in dilution buffer TBS (10 mM Tris-HCl pH 8, 10 mM KCl, 100 mM NaCl, 10 mM imidazole) and 1% BSA. In each binding reaction, 200 μl of p12 was incubated with 4 μl of CA-coated lipid nanotubes or 1 μl of CA-coated beads, for 2 hours at room temperature with gentle agitation. The samples were then layered on top of a 2 ml cushion of 40% (w/v) sucrose in TBS and centrifuged at 34,000 g for 1 hour at 4°C. The supernatants were then aspirated and the pellets were resuspended in 40 μl of protein loading buffer. His-tagged CA and p12 in the pellet fractions were detected by immunoblotting.

### Immunoblotting

Proteins were separated by SDS polyacrylamide gel electrophoresis (SDS-PAGE) and transferred onto polyvinylidene fluoride (PVDF) membranes (Milipore). The primary antibodies used in immunoblotting are included in S4 Table. Unless specifically stated, the rabbit antibody raised against a p12 peptide antigen was used to probe for p12. Goat anti-rabbit IRDye800CW (LI-COR, 1:5000), goat anti-mouse IRDye680RD (LI-COR, 1:5000) and goat anti-mouse HRP (Pierce, 1:10,000) were used as secondary antibodies. Immuno-complexes were detected either using the Li-cor Odyssey imaging and quantitation system (LI-COR Bioscience) or hyperfilm processing with Fijifilm FPM-3800A developer.

### Co-immunoprecipitation (Co-IP) assays with viral lysates

Titres of VLPs carrying myc-tagged p12 were normalised based on CA amount. Aliquots of VLPs were concentrated by centrifugation through a 20% (w/v) sucrose cushion for 2 h at 10,000 g, 4°C and re-suspended in protein loading buffer. Samples were then analysed by immunoblotting together with a panel of serially-diluted purified N-MLV CA of known concentration. CA amounts were interpolated from the mean band intensity measurements.

For Co-IP assays, myc-tagged p12 VLPs were concentrated by centrifugation through sucrose as above and re-suspended in 1% formaldehyde in PBS for 20 min at room temperature. The cross-linking reaction was then quenched for 10 min by the addition of Tris-HCl pH 7.5 to a final concentration of 250 mM. The cross-linked VLPs were again spun through sucrose and re-suspended in RIPA lysis buffer (Thermo Fisher Scientific) supplemented with protease inhibitors (Roche). Lysis was facilitated by sonication, x7 pulses, 30 s ON and 30 s OFF, in an ice bath (Decon FS100). After sonication, 4 mM MgCl_2_ and Pierce universal nuclease (Thermo Fisher Scientific) were added to the viral lysates. The lysates were then normalised based on previously-estimated CA amounts and incubated overnight with Protein G Dynabeads (Thermo Fisher Scientific) carrying immobilised anti-myc antibody 9E10 [10]. After approximately 16 h, the beads were washed three times, re-suspended in protein loading buffer, boiled and analysed by immunoblotting with anti-p12 and anti-CA antibodies (S4 Table).

### Single-cycle infectivity assays

HeLa or D17 cells were seeded in 24-well plates (Corning) at densities of 2.5x10^4^ cells/well and 2x10^4^ cells/well, respectively. Cells were infected 24 h later, with WT and mutant VLPs normalised on their RT activity and incubated at 37°C for 72 h. Cells were then lysed in Tropix Lysis buffer (Thermo Fisher Scientific) and frozen at -20°C. To measure LacZ activity, cell lysate was mixed with Tropix galactostar reaction mixture (Thermo Fisher Scientific) and luminescence was measured for 1 h at 10 min intervals on a Tecan Safire plate reader.

Absolute infectious titres of VLPs for microscopy assays were determined by X-gal staining of infected HeLa cells. HeLa cells were seeded in 12-well plates (Corning) at a density of 5x10^4^ cells/well and infected 24 h later with a 10-fold dilution series of VLPs by spinoculation (1600 g, 2 h, 16°C). Cells were then incubated at 37°C for 30 min prior to replacement of media with warm serum-supplemented DMEM. After 72 h, cells were washed in PBS and fixed for 10 min with 2% formaldehyde and 0.2% glutaraldehyde. Staining was performed overnight at 37°C in PBS with 0.4 mg/ml X-gal (5-bromo-4-chloro-3-indolyl- beta-D-galactopyranoside (Sigma), 4 mM K3Fe(CN)6 (Sigma), 4mM K4Fe(CN)6ˑ3H2O (Sigma), 2mM MgCl_2_ (Thermo). The number of blue LacZ-expressing colonies were counted using a light microscope (Olympus) and the viral titre calculated.

### Immunofluorescence

To synchronise the cell cycle of HeLa cells, 2 μg/ml of aphidicolin (Sigma) was added for 15–16 h. The cells were then washed and incubated in normal media for 0.5–1.5 h before re-seeding on 13 mm coverslips. Cells were incubated at 37°C for 7–8 h before the addition of 2 μg/ml of aphidicolin for a further 14–15 h. Cells were then washed and allowed to recover for 30 min in normal media before infection with VLPs by spinoculation (1600 g, 2 h, 16°C) at a MOI of <1. The cells were incubated at 37°C for 30 min before replacement of media with fresh serum-supplemented DMEM and returned to the incubator for 10 h. The cells were then washed twice with PBS and fixed with 4% paraformaldehyde for 5 min at room temperature followed by ice-cold methanol at -20 ^o^C for 5 min. Cells were subsequently permeablised with 0.5% saponin (Sigma) in PBS for 30 min and blocked in 5% normal donkey serum (NDS, Source Bioscience) and 0.5% saponin in PBS for at least 1 h. Cells were then incubated with rabbit anti-p12 (custom generated against a p12 peptide by Cambridge Research Biochemicals) and rat anti-CA (CRL-1912, ATCC) antibodies diluted in 1% NDS and 0.5% saponin in PBS for 1 h, at RT. Coverslips were then washed three times with PBS and incubated for another hour with anti-mouse and anti-rat secondary antibodies conjugated to Alexa Fluor 594 and 488 dyes (Abcam, ab150064 and ab150117), diluted in 1% NDS PBS and 0.5% saponin in PBS. Coverslips were finally washed three times and mounted in Prolong Gold media with DAPI (Thermo Fisher Scientific) on glass slides (Menzel-Gläser). Clear nail varnish was used for sealing the coverslips. Immunostained cells were visualised on a SP5 inverted confocal microscope (Leica) using a HCX PL APO CS 100.0x1.46 OIL (Leica) objective. Image analysis was performed using FIJI https://fiji.sc. For co-localisation analysis, three-dimensional stacks were projected in Z by summation to generate two-dimensional images. The DAPI channel was then pre-processed by median filtering to suppress noise and grey-level thresholded using the Huang algorithm to generate binary mask images. Interphase nuclei and artefacts were removed by specifying object area and circularity thresholds in the Particle Analyzer.

Co-localisation analysis of p12 and CA puncta was then performed using a custom plug-in produced in-house–further details and complete source code are available online (https://bitbucket.org/djpbarry/particletracker/wiki/Particle%20Mapper). Briefly, puncta in one channel were detected using a Laplacian-of-Gaussian based detection scheme (referred to as ‘Blobs’ detection mode within the plug-in)–puncta exhibiting an intensity below a pre-specified threshold were discarded. Co-localisation was then evaluated by determining how many puncta in the first channel are co-incident with local intensity maxima above a second threshold exist in the second channel.

HeLa cells stably expressing GST-p12 were fixed in 4% paraformaldehyde and methanol as described above and then stained with rabbit anti-p12 or anti-GST (Abcam, ab19256) primary antibodies and the Alexa Fluor 594 secondary antibody. The samples were visualised on SP5 using a 100X (1.46NA) objective and analysed on ImageJ 1.49v.

### Biochemical fractionation

GST-p12 proteins were expressed in 293T cells from pCAGGS/GST-derived plasmids by transient transfection using Turbofect (Thermo Fisher Scientific). Cell media was changed 24 h after transfection, and cells were incubated overnight at 37°C, before being harvested, counted, pelleted (500 g, 5 min) and snap frozen in dry ice/ethanol. Biochemical fractionation of cells was performed essentially as described in [14]. Briefly, cell pellets thawed on ice were resuspended at 2.5× 10^7^ cells/ml in buffer 1 (10 mm HEPES pH 7.9, 10 mm KCl, 1.5 mm MgCl_2_, 10% glycerol, 0.34 m sucrose, 1 mm DTT and protease inhibitors) and then supplemented with 0.1% Triton-X-100. Cells were incubated on ice for 5 min and nuclei (P1) were collected by centrifugation at 1300 g, 5 min, 4°C. The supernatant (S1) was clarified by further centrifugation (5 min, 20 000 × ***g***, 4°C) to collect the cytosolic supernatant fraction (S2). The P1 nuclei were washed once with buffer 1 and then lysed for 30 min in buffer 2 (3 mM EDTA, 0.2 mM EGTA and 1 mM DTT, protease inhibitors). The chromatin (P3) and soluble nuclear (S3) fractions were separated by centrifugation (5 min, 1700 g, 4°C). The S2, S3 and P3 fractions were analysed by immunoblotting.

### Pull-down assays using glutathione-sepharose beads

GST-p12 proteins were expressed in 293T cells from pCAGGS/GST-derived plasmids by transient transfection using Turbofect (Thermo Fisher Scientific). Approximately 24 h after transfection, cells were changed into fresh media containing either 400 ng/ml nocodazole (Sigma) or 2 μg/ml aphidicolin (Sigma) and incubated overnight at 37°C. The synchronisation of the cell cultures after drug treatment was assessed by propidium iodide staining and cell cycle phase analysis by flow cytometry. Cells to be used in pull down assays were washed and lysed in 20 mM Tris pH 8.0, 300 mM NaCl, 1 mM EDTA, 10% Glycerol, 1% Triton X-100, protease inhibitors (Roche), phosphatase inhibitors (Merck). Lysis was facilitated by passing the lysates through a 19-gauge needle x7 and sonication, x7 pulses, 30 s ON and 30 s OFF, in an ice bath (Decon FS100). After sonication, the lysates were supplemented with 4 mM MgCl_2_ and Pierce universal nuclease (Thermo Fisher Scientific) and incubated for a further 1 h at 4°C. The lysates were then clarified by centrifugation and normalised based on total protein concentration using the Pierce BCA Protein Assay kit (Thermo Fisher Scientific). 0.5 ml aliquots of lysates at 1.5–3 mg/ml were incubated with glutathione-sepharose beads (100 μl/reaction of a 50% slurry) (GE Healthcare) for 3 h at 4°C with end-over-end rotation. The beads were then washed three times in 20 mM Tris pH 8.0, 300 mM NaCl, 1 mM EDTA, 10% Glycerol, 0.1% Triton X-100, protease inhibitors and re-suspended in 50 μl of x2 protein loading dye for SDS-PAGE and immunoblotting. Silver-staining was performed using the SilverQuest kit (Thermo Fisher Scientific). ProQ diamond and Sypro ruby staining (Thermo *Fisher Scientific*) of gels was visualised and quantified on a ChemiDoc imaging system (Bio-Rad).

For SILAC-MS experiments, 293T cells were grown is media containing light (R0K0, ^12^C-Arginine and ^12^C-Lysine), medium (R6K4, ^13^C-Arginine and Lysine-4,4,5,5-d4) or heavy (R10K8, ^13^C+^15^N-Arginine and ^13^C+^15^N-Lysine) versions of L-Arginine and L-Lysine amino acids (Pierce) for a minimum of 6 doublings before transient expression of GST-p12 proteins. After performing the glutathione-sepharose pull-down assays equivalent volumes of the beads eluates were pooled and submitted to the Bristol University Proteomics Facility for RP nano-LC-MS/MS analysis. This analysis was performed on an LTQ-Orbitrap Velos mass spectrometer in line with a Dionex Ultimate 3000 nanoHPLC system as described in [53].

To identify phosphopeptides of GST-p12, bead eluates were separated by SDS-PAGE and stained using SimplyBlue safe stain (Thermo Fisher Scientific). The band corresponding to GST-p12 was then excised and submitted for nano-LC-MS/MS analysis as described in [54], but without phosphopeptides enrichment. The probability-based phosphoRS algorithm was used for assigning phosphorylation sites in the detected peptides [32].

### Pull-down assays using DNA-coated cellulose beads

GST-p12 and IN-HA were expressed in 293T cells from pCAGGS/GST and pCMV4/HA-derived plasmids respectively, by transient transfection using Turbofect (Thermo Fisher Scientific). Approximately 24 h after transfection, cells were changed into fresh media and incubated overnight at 37°C. Cells were then washed and lysed in 20 mM Tris-HCl pH 8.0, 300 mM NaCl, 1 mM EDTA, 10% Glycerol, 1% Triton X-100, protease inhibitors. Lysis was facilitated by passing the lysates through a 19-gauge needle x7 and sonication, x7 pulses, 30 s ON and 30 s OFF, in an ice bath (Decon FS100). After sonication, the lysates were supplemented with 4 mM MgCl_2_ and 1 mM DTT, and incubated for a further 1 h at 4°C. The lysates were then clarified by centrifugation and diluted 1:3 in 20 mM Tris-HCl pH 8.0, 10% glycerol, 4 mM MgCl_2_ and 1 mM DTT. The lysates were then normalised based on total protein concentration using the Bradford protein assay (Bio-Rad). 0.7 ml aliquots of lysates at 0.7 mg/ml were incubated with calf thymus DNA-coated cellulose beads (100 μl/reaction of a 50% slurry) (Sigma) for 1 h at 4°C with end-over-end rotation. The beads were then washed three times in 20 mM Tris pH 8.0, 100 mM NaCl, 0.33 mM EDTA, 10% Glycerol, 0.33% Triton X-100, 4 mM MgCl_2_ and 1 mM DTT and re-suspended in 50 μl of x2 protein loading dye for SDS-PAGE and immunoblotting.

### Kinase inhibition

GST-p12 proteins were expressed in 293T cells from pCAGGS/GST-derived plasmids by transient transfection using Turbofect (Thermo Fisher Scientific). Approximately 24 h after transfection, cells were changed into fresh media containing 400 ng/ml nocodazole (Sigma) and incubated overnight at 37°C. Cells were then changed into media containing 400 ng/ml nocodazole, 10 μM MG132 (Sigma) and either 40 mM LiCl (Sigma), 75 μM roscovitine (Sigma) or 40 μM kenpaullone (Sigma), for 3.5 h. Cells were immediately washed and lysed for glutathione-sepharose pull-down assays as described above.

### Biolayer interferometry measurements

The binding of PFV *h*CBS and p12 CTD peptides to recombinant H3.3 poly-nucleosomes (Active Motif) was measured using biolayer interferometry on an Octet RED system (Pall ForteBio Corp). The peptides were synthesised with a N-terminal biotin tag and GGGG linker. The Mo-MLV, FeLV and GaLV p12 CTD peptide sequences used are highlighted in bold in Fig 5C. A negative control was included that contained an R66A change in the Mo-MLV p12_M63I CTD peptide. Mo-MLV phos p12_M63I CTD was also synthesised with a phosphorylated S61 residue. The *h*CBS (positive control) sequence used corresponds to the CBS of PFV Gag (NQGGYNLRPRTYQPQRYG). The biotinylated peptides were loaded onto streptavidin biosensors (Pall ForteBio Corp) at 0.5 μg/ml for 120 s in 10 mM HEPES pH 7.5, 150 mM NaCl, 0.005% (v/v) Tween 20. Following a buffer wash, the biosensors were incubated with serially-diluted nucleosome preparations (~0.5–250 nM) for 900–5400 s to measure association. Experiments were performed at 25°C and sample plates were agitated at 1000 rpm. Equilibrium binding amplitudes were determined by exponential least squares curve fitting of the association phase. The estimated binding amplitudes were then plotted against approximate nucleosome concentrations on GraphPad Prism 7. Equilibrium binding constant (K_d_) and maximum specific binding (B_max_) were calculated by fitting the data to a non-linear regression model. Experiments were performed at least in duplicate and the data pooled for fitting. For competition assays, soluble non-biotin tagged peptides were added to the nucleosomal arrays at 100 μM to inhibit nucleosome binding prior to recording BLI measurements with biotinylated peptide immobilised on sensor probes. The non-biotin tagged peptide sequences were: *h*CBS—NQGGYNLRPRTYQP; *h*CBS_L539A/R540A - NQGGYNAAPRTYQP; p12_WT—DPSPMASRLRGRREPPY; p12_R66A - DPSPMASALRGRREPPY.

## Supporting information

S1 Fig Estimating the stoichiometry of the p12-CA interaction. **(Links to Fig 1).** A 16-fold molar excess of purified p12_WT was incubated with His-tagged WT N-MLV CA immobilised on DGS-NTA-containing lipid nanotubes. The p12-CA complexes were separated from free p12 by centrifugation through a sucrose cushion and the pelleted fraction was analysed by immunoblotting for CA (anti-His) and p12 (mouse anti-p12). The blots were visualised and the median intensities of the p12-bands were estimated using a Li-cor Odyssey. (A) Representative immunoblot of a p12 input sample (serially diluted, 1/10-1/160) and pelleted fractions from two experimental replicates, R1 (lane 6) and R2 (lane 7). (B) Standard curve generated from the estimated median intensities of the serially-diluted p12 input. (C) Calculation of the amount of p12 pelleted by CA-nanotubes with reference to the standard curve to determine the stoichiometry of p12 molecules to CA monomers.
(TIF)

S2 Fig SILAC results showing no significant difference in protein complexes of Mo-MLV GST-p12_mut14 compared to GST-p12_WT. **(Links to Fig 4).** GST, Mo-MLV GST-p12_mut14 or GST-p12_WT were transiently-expressed in 293T cells cultured in light (L), medium (M) or heavy (H) SILAC media, respectively. GST-protein complexes were precipitated from mitotic cell lysates and analysed by LC-MS/MS. (A) To identify proteins enriched in the GST-p12_WT (H) sample relative to the GST-p12_mut14 (M) sample, log_2_(H/M) silac ratios of each set of MS hits (FDR <5%) from replicates (R1 and R2) were plotted as a frequency distribution. Mean and SD of each distribution was estimated by fitting to a normal distribution curve (R^2^ ≥ 0.98). (B) MS hits were grouped based on the number of SDs from the mean. There was no overlap between the replicates until the threshold was lowered to ≥1 SD from the mean. The selection criteria for significant enrichment was ≥ 2.58 SDs from the mean in both replicates.
(TIF)

S3 Fig The M63I mutation confers mitotic chromatin binding of GST-tagged Mo-MLV p12. **(Links to Fig 5).** Representative confocal microscopy images showing localisation of stably-expressed full-length GST-p12 mutants (top panels) and GST-p12 CTD fragments (bottom panels) in HeLa cells. p12 mutations: M63I, G49R/E50K, D25A/L-dom (carrying alanine substitutions of the PPPY motif as well as D25A, which disrupts clathrin binding), R66A and +*h*CBS. Cells were stained for p12 (anti-p12, red or anti-GST, green), and DNA (DAPI, blue). White boxes indicate mitotic cells.
(TIF)

S4 Fig The chromatin interactomes of GST-p12_M63I and GST-p12+*h*CBS/Mut14 largely overlap. **(Links to Fig 8).** GST-p12_M63I/Mut14, GST-p12_M63I or GST-p12+*h*CBS/Mut14 were transiently-expressed in 293T cells cultured in light (L), medium (M) or heavy (H) SILAC media, respectively. GST-protein complexes were isolated from mitotic cell lysates and analysed by LC-MS/MS. Cellular proteins enriched in the GST-p12_M63I (M) sample relative to the GST-p12_M63I/mut14 (L) sample were identified from the log_2_(M/L) silac ratios of two biological replicates, as described in Figs 4B and 8A. GST-p12+*h*CBS/Mut14 chromatin interactants were similarly identified from the log_2_(H/L) ratios of the mass-spec hits. The Venn diagram shows the overlap between the 73 proteins enriched in GST-p12_M63I samples (orange shading) and the 80 proteins enriched in the GST-p12+*h*CBS/Mut14 samples (purple shading). The proteins enriched in both samples (pink shading) are listed in the table.
(TIF)

S1 Table Results from the first SILAC-MS analysis. GST, Mo-MLV GST-p12_mut14 or GST-p12_WT were transiently-expressed in 293T cells cultured in light (R0/K0), medium (R6/K4) and heavy (R10/K8) SILAC media, respectively. Mitotic cell lysates were subsequently used for glutathione-sepharose bead pull-down assays. Bead eluates were pooled in a 1:1:1 ratio and then analysed by LC-MS/MS. The details of MS hits which showed log_2_(H/L) ratios >2.58 SDs from the mean, in both biological replicates (R1 and R2), are shown.
(XLSX)

S2 Table Results from the second SILAC-MS analysis. Mo-MLV GST-p12_M63I or GST-p12_WT were transiently-expressed in 293T cells cultured in light (R0/K0) or medium (R6/K4) SILAC media, respectively. Mitotic cell lysates were subsequently used for glutathione-sepharose bead pull-down assays. Bead eluates were pooled in a 1:1 ratio and then analysed by LC-MS/MS. The details of MS hits which showed log_2_(L/M) ratios >2.58 SDs from the mean, in both biological replicates (R1 and R2), are shown.
(XLSX)

S3 Table Primers used for site directed mutagenesis. (TIF)

S4 Table Primary antibodies used in immunoblotting. (TIF)
