# Supplementary figures and images for: Murine leukemia virus p12 tethers the capsid-containing pre-integration complex to chromatin by binding directly to host nucleosomes in mitosis
Source: PLoS Pathog. 2018 Jun 15;14(6):e1007117. doi: 10.1371/journal.ppat.1007117 (PMC6021111; doi:10.1371/journal.ppat.1007117)

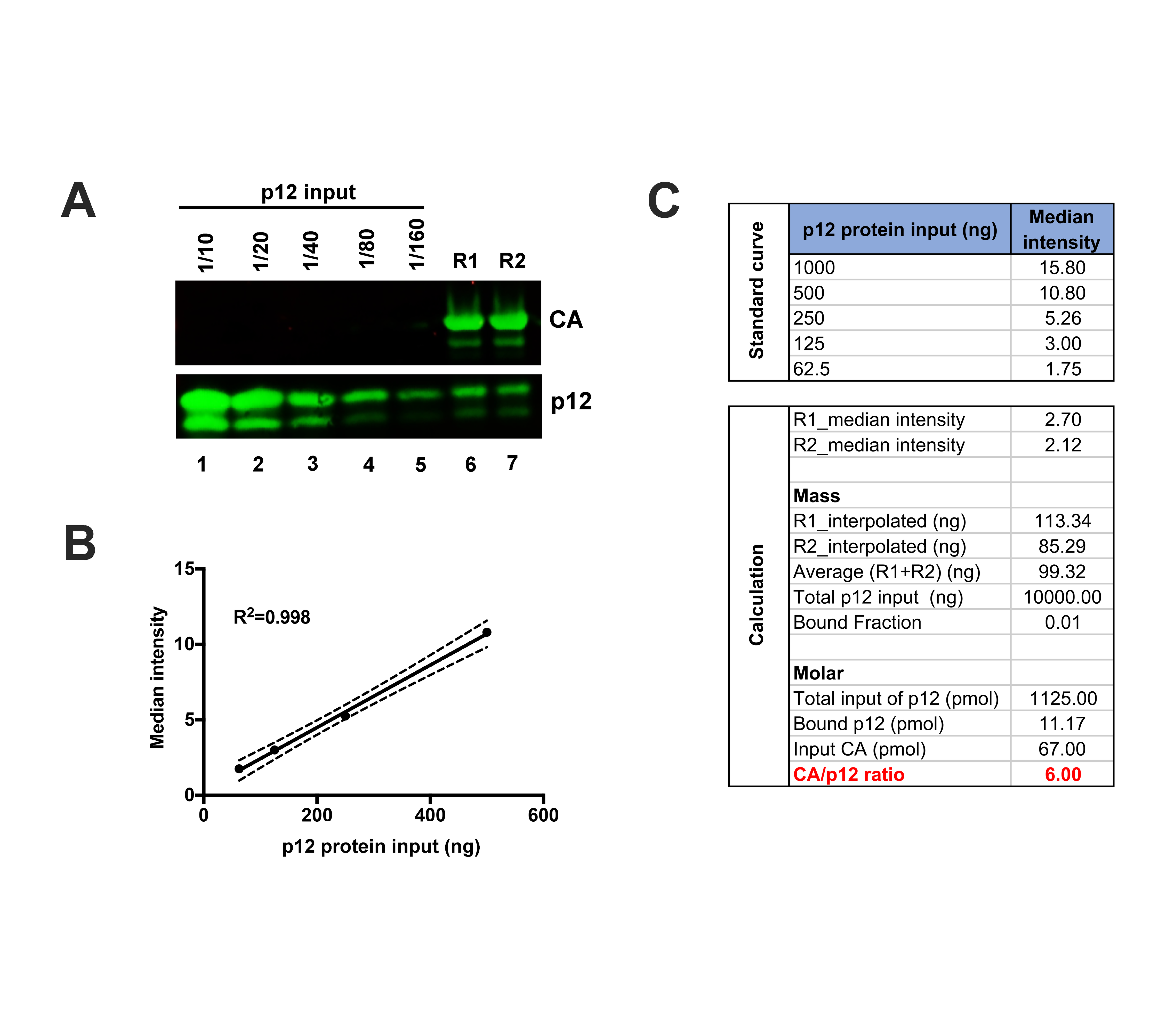

Supplement: S1 Fig — (Links to Fig 1). A 16-fold molar excess of purified p12_WT was incubated with His-tagged WT N-MLV CA immobilised on DGS-NTA-containing lipid nanotubes. The p12-CA complexes were separated from free p12 by centrifugation through a sucrose cushion and the pelleted fraction was analysed by immunoblotting for CA (anti-His) and p12 (mouse anti-p12). The blots were visualised and the median intensities of the p12-bands were estimated using a Li-cor Odyssey. (A) Representative immunoblot of a p12 input sample (serially diluted, 1/10-1/160) and pelleted fractions from two experimental replicates, R1 (lane 6) and R2 (lane 7). (B) Standard curve generated from the estimated median intensities of the serially-diluted p12 input. (C) Calculation of the amount of p12 pelleted by CA-nanotubes with reference to the standard curve to determine the stoichiometry of p12 molecules to CA monomers. (TIF) [file ppat.1007117.s001.tif]

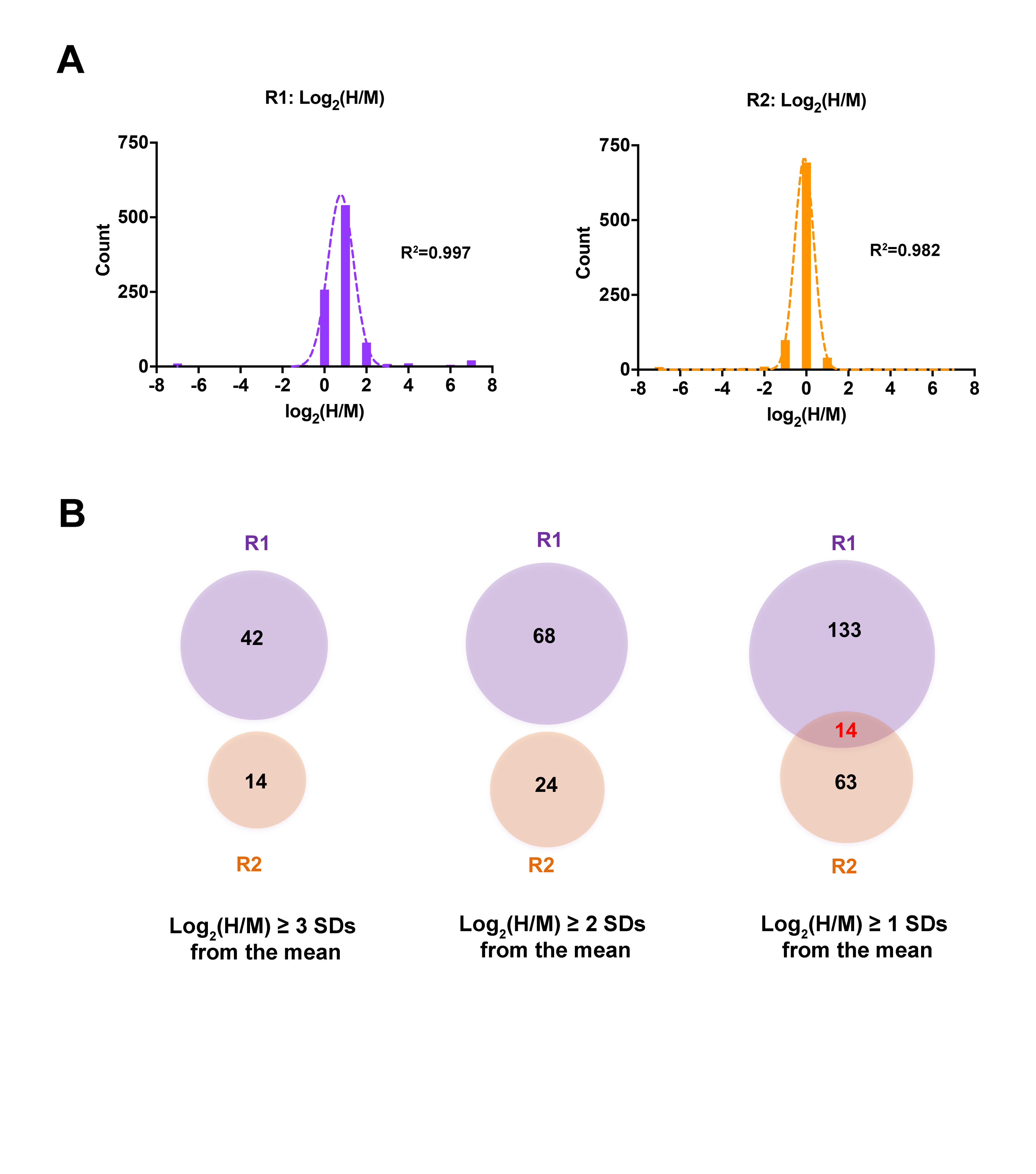

Supplement: S2 Fig — (Links to Fig 4). GST, Mo-MLV GST-p12_mut14 or GST-p12_WT were transiently-expressed in 293T cells cultured in light (L), medium (M) or heavy (H) SILAC media, respectively. GST-protein complexes were precipitated from mitotic cell lysates and analysed by LC-MS/MS. (A) To identify proteins enriched in the GST-p12_WT (H) sample relative to the GST-p12_mut14 (M) sample, log2(H/M) silac ratios of each set of MS hits (FDR <5%) from replicates (R1 and R2) were plotted as a frequency distribution. Mean and SD of each distribution was estimated by fitting to a normal distribution curve (R2 ≥ 0.98). (B) MS hits were grouped based on the number of SDs from the mean. There was no overlap between the replicates until the threshold was lowered to ≥1 SD from the mean. The selection criteria for significant enrichment was ≥ 2.58 SDs from the mean in both replicates. (TIF) [file ppat.1007117.s002.tif]

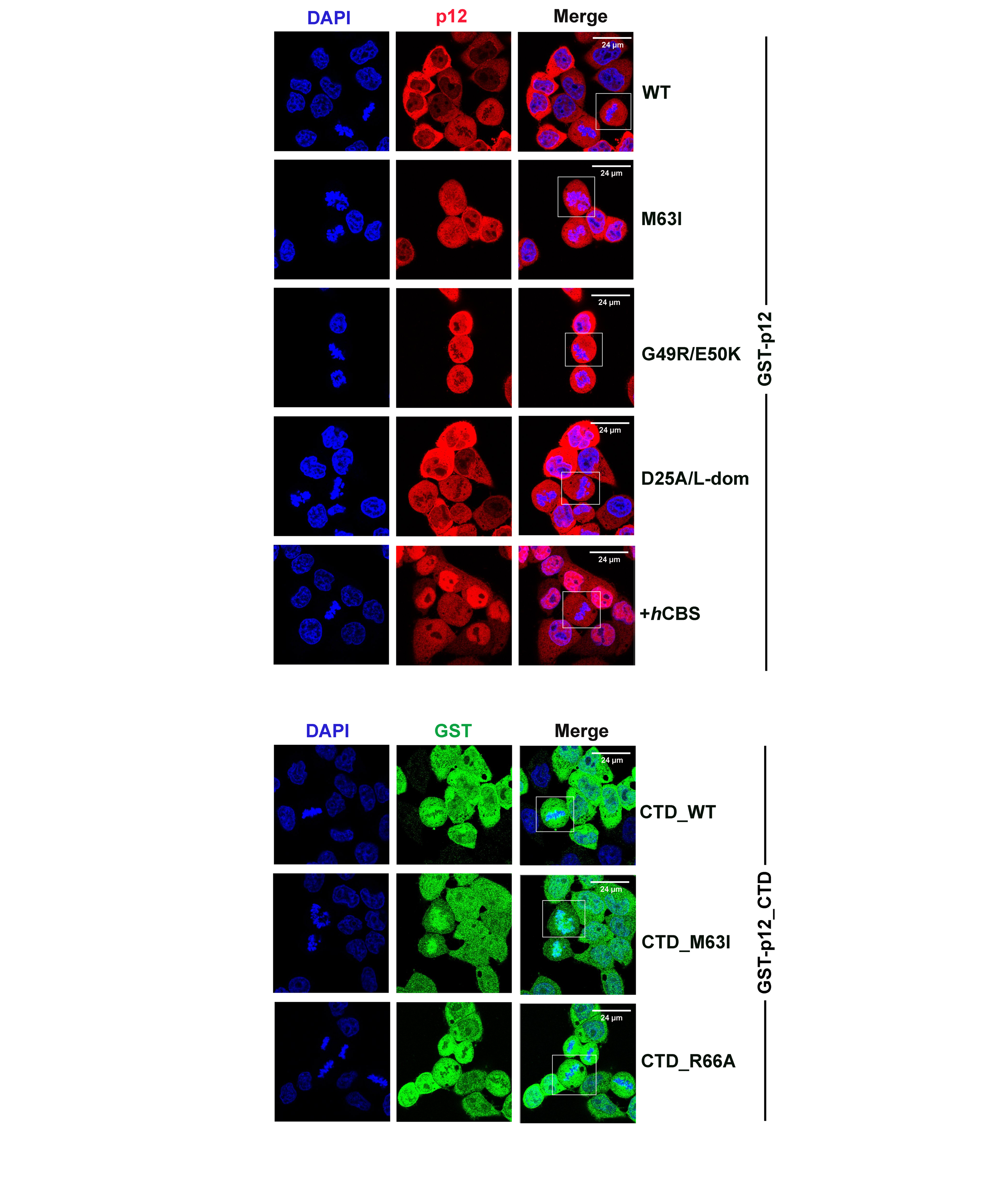

Supplement: S3 Fig — (Links to Fig 5). Representative confocal microscopy images showing localisation of stably-expressed full-length GST-p12 mutants (top panels) and GST-p12 CTD fragments (bottom panels) in HeLa cells. p12 mutations: M63I, G49R/E50K, D25A/L-dom (carrying alanine substitutions of the PPPY motif as well as D25A, which disrupts clathrin binding), R66A and +hCBS. Cells were stained for p12 (anti-p12, red or anti-GST, green), and DNA (DAPI, blue). White boxes indicate mitotic cells. (TIF) [file ppat.1007117.s003.tif]

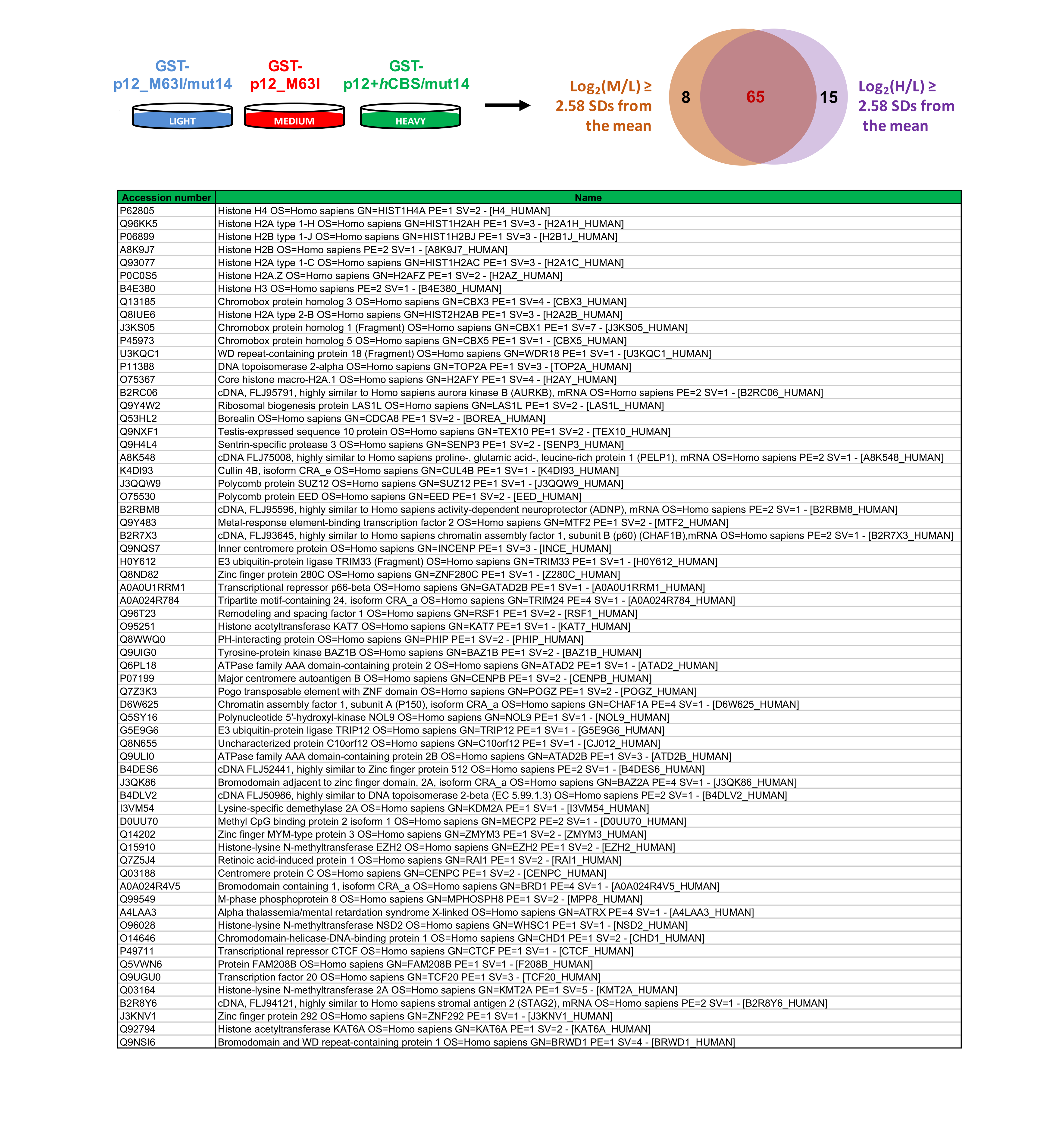

Supplement: S4 Fig — (Links to Fig 8). GST-p12_M63I/Mut14, GST-p12_M63I or GST-p12+hCBS/Mut14 were transiently-expressed in 293T cells cultured in light (L), medium (M) or heavy (H) SILAC media, respectively. GST-protein complexes were isolated from mitotic cell lysates and analysed by LC-MS/MS. Cellular proteins enriched in the GST-p12_M63I (M) sample relative to the GST-p12_M63I/mut14 (L) sample were identified from the log2(M/L) silac ratios of two biological replicates, as described in Figs 4B and 8A. GST-p12+hCBS/Mut14 chromatin interactants were similarly identified from the log2(H/L) ratios of the mass-spec hits. The Venn diagram shows the overlap between the 73 proteins enriched in GST-p12_M63I samples (orange shading) and the 80 proteins enriched in the GST-p12+hCBS/Mut14 samples (purple shading). The proteins enriched in both samples (pink shading) are listed in the table. (TIF) [file ppat.1007117.s004.tif]

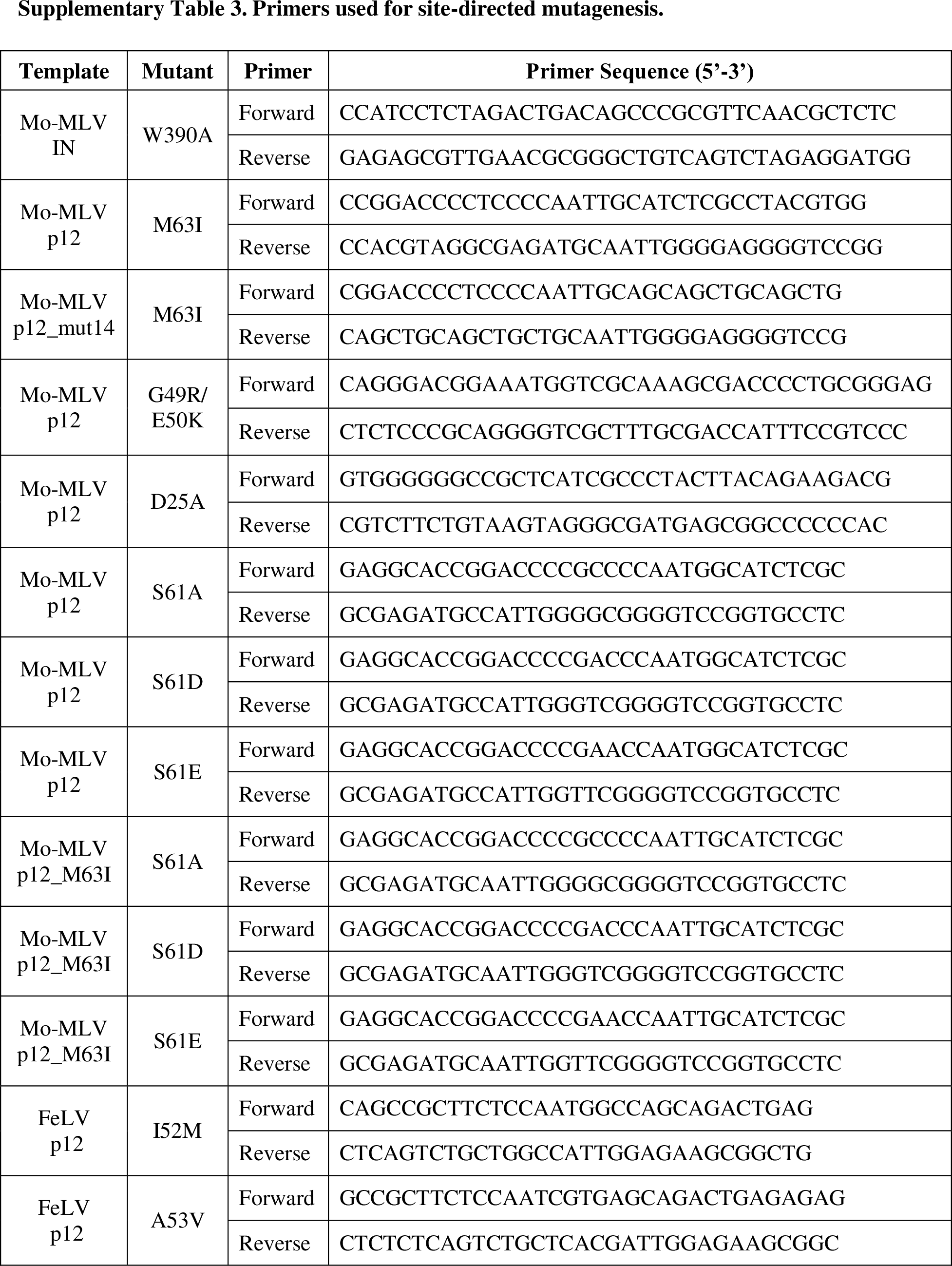

Supplement: S3 Table — (TIF) [file ppat.1007117.s007.tif]

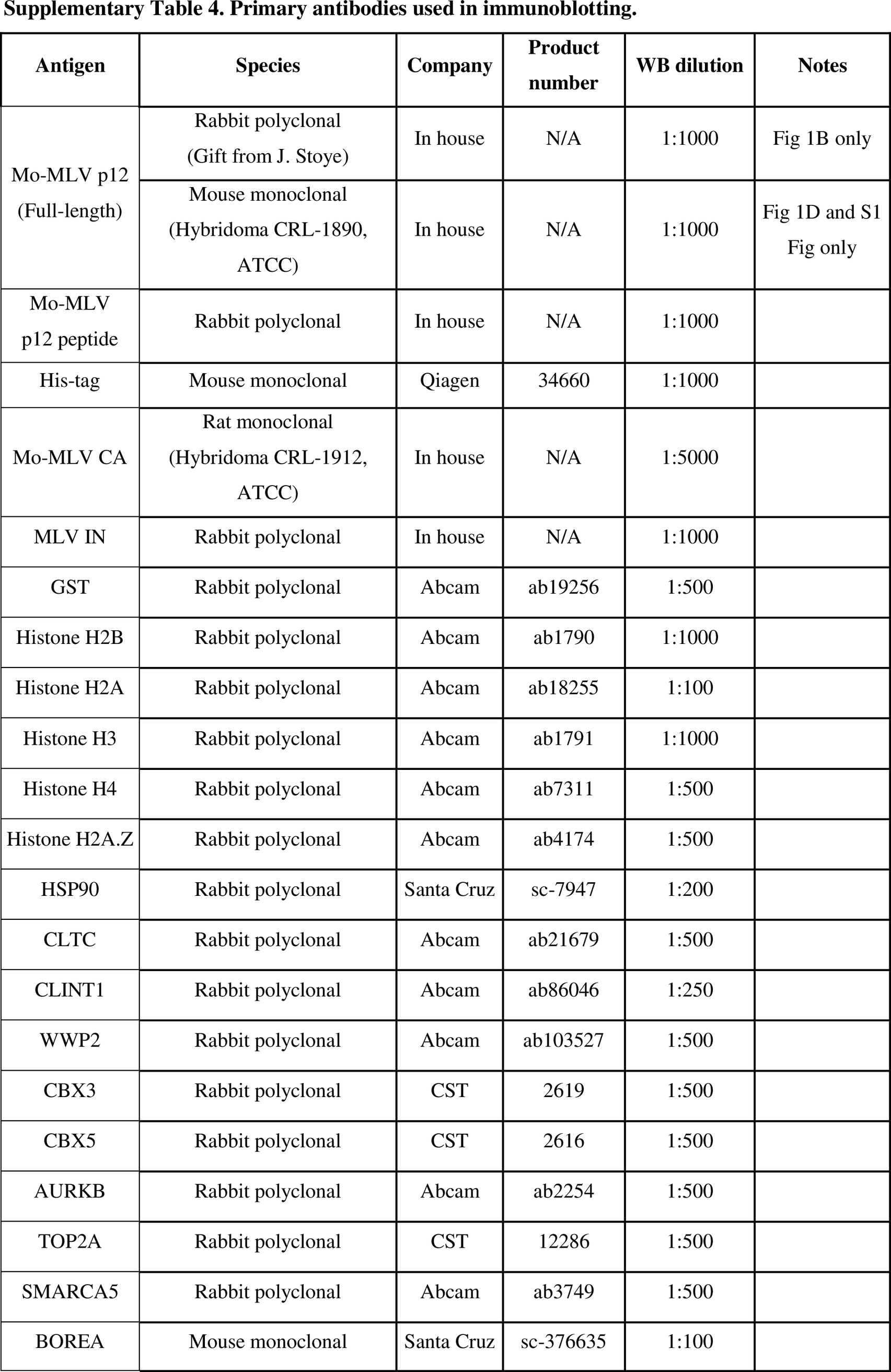

Supplement: S4 Table — (TIF) [file ppat.1007117.s008.tif]
